# Bronze Age Raw Material Hoard from Greater Poland: Archaeometallurgical Study Based on Material Research, Thermodynamic Analysis, and Experiments

**DOI:** 10.3390/ma17010230

**Published:** 2023-12-31

**Authors:** Aldona Garbacz-Klempka, Marcin Piękoś, Janusz Kozana, Małgorzata Perek-Nowak, Marta Wardas-Lasoń, Patrycja Silska, Mateusz Stróżyk

**Affiliations:** 1 Faculty of Foundry Engineering, AGH University of Krakow, Reymonta 23 St., 30-059 Krakow, Poland; mpiekos@agh.edu.pl (M.P.); jkozana@agh.edu.pl (J.K.); 2 Faculty of Non-Ferrous Metals, AGH University of Krakow, Mickiewicza 30, 30-059 Krakow, Poland; mperek@agh.edu.pl; 3 Faculty of Geology, Geophysics and Environmental Protection, AGH University of Krakow, Mickiewicza 30, 30-059 Krakow, Poland; mw@agh.edu.pl; 4 Department of Prehistoric Archaeology, Archaeological Museum in Poznań, Wodna 27 St., 61-781 Poznań, Poland; patrycja.silska@muzarp.poznan.pl (P.S.); mateusz.strozyk@muzarp.poznan.pl (M.S.)

**Keywords:** archaeometallurgy, copper, copper alloys, casting, CALPHAD, computer modelling, ED-XRF, SEM-EDS, EBSD, Late Bronze Age, metalwork, metal hoards, recycling, hoarding, deposits

## Abstract

Hoard finds from the Bronze Age have appeared all over Europe, prompting questions about their functions (either as raw materials for recycling or votive objects). The hoard trove of raw materials from Przybysław in Greater Poland is an interesting example of a discovery that is related to the foundry activities of Late Bronze Age and Early Iron Age communities (c. 600 BC). The deposit consists of fragments of raw materials that were damaged end products intended for smelting. The research included the characterisation of the material in terms of the variety of the raw materials that were used. The individual elements of the hoard were characterised in terms of their chemical compositions, microstructures, and properties. A range of modern instrumental research methods were used: metallographic macroscopic and microscopic observations by optical microscopy (OM), scanning electron microscopy (SEM), chemical-composition analysis by X-ray fluorescence spectroscopy (ED-XRF), X-ray microanalysis (EDS), and detailed crystallisation analysis by electron microscopy with an emphasis on electron backscatter diffraction (EBSD). As part of this study, model alloys were also prepared for two of the selected chemical compositions, (i.e., CuPbSn and CuPb). These alloys were analysed for their mechanical and technological properties. This research of the hoard from Przybysław (Jarocin district, Greater Poland) has contributed to the recognition and interpretation of the function and nature of the hoard by using modern research and modelling methods as a cultic raw material deposit.

## 1. Introduction

Hoard finds from the Bronze Age and the Early Iron Age have appeared all over Europe, prompting questions about their functions (either as hoard troves of votive objects or as raw materials for recycling that were linked to technological processes) [1].

Hoard is a concept that encompasses the accumulation of whole or fragmentary artfacts for ritualistic (ritual gifts) or non-ritualistic purposes as well as for permanent or temporary storage and deposition. The core of the problem is the functional classification of hoards [2,3,4,5,6].

In prehistoric societies, the technological operations that led from the receipt of the raw materials to the finished product were highly ritualised [7,8].

As noted in those hoards that have been reduced to fragments of destroyed wares, traces of technological operations and their successive operational sequences (chaîne opératoire) of production and destruction can be helpful when interpreting destruction and scrapping as a ritual practice [4].

Modern instrumental research techniques [9,10,11,12,13] and computer-aided process analysis methods are important for understanding prehistoric metalworking [14,15,16,17,18]. The study of the prehistoric metallurgical and foundry processes of the Late Bronze Age and Early Iron Age focuses on raw materials, ores, analyses of semi-finished products, slags, and finished products. Traces of old technologies are also preserved in the form of casting moulds and gating systems, along with foundry workshop equipment such as tools, jets as furnace remains, or crucibles [19,20]. Earthen deposits also bear witness to these periods’ manufacturing activities [21].

The production process of bronze products involved a number of stages, starting with the acquisition of raw materials, and the primary production of metal from ore (i.e., copper smelting) and secondary production (i.e., used of bronze scrap, melting and the possible introduction of alloying additives), followed by casting and processing [22,23]. Primary materials smelted from ores were used in the form of copper or tin ingots: discs, bars, and rods [24,25,26,27,28,29]. The distribution of the raw materials was based on the trade in raw materials as well as in semi-finished and finished products. Raw material was also obtained from recycling by smelting secondary materials, including the remnants of their own technological waste (so-called circulating scrap) as well as damaged or defective products [19,22,30,31]. The changing elemental compositions that were recorded in cast products are evidence of technological progress and an increasing awareness of the influence of basic tin- and lead-alloying elements on product properties.

Casting techniques have been very important in the development of manufacturing techniques with such processes as metal technology (melting, refining and alloying) [19,22], mould preparation technology [18,32,33,34] and finishing processes.

Assessing the degree of the specialisation of a foundry, which is visible in the designs of its moulds and gating systems and the finishing techniques that were used, is possible by observing the preserved evidence in the forms of moulds and castings using modern research methods and computer-aided processes [18,33,34,35]. Any traces that were left by the after-treatment and any defects that were caused by deformation and thermal fatigue during the forming process can lead to attempts to reconstruct sequential operations that indicate the technology that was used. These traces can be used to define the tools and manufacturing techniques that were used as well as the sequences of subsequent operations [36].

In the study of the Bronze Age, new insights into the phenomenon of the deposition of metal objects have emerged in recent decades. They are no longer merely seen as storerooms for merchants or as places for hiding wealth during times of peril but are beginning to be considered as culturally sanctioned, social phenomena [4,37,38,39,40]. Analyses include the chronological and regional variations in the deposition practices, the compositions and conditions of the finds, the distributions of the deposited objects in relation to other archaeological finds, and the locations of the finds in the landscape [41,42,43,44,45,46,47]. However, the technological aspect has been underrepresented thus far in the interpretation of metal hoards, whereas the data from metallurgical analyses can also be important in interpreting the functions and natures of such hoards.

This research of the hoard from Przybysław (Jarocin district, Greater Poland) aims to fill this gap and contribute to the recognition of the role of the hoard by using modern research and modelling methods. The above-mentioned hoard became part of the collection of the Mielżyński Museum in Poznań in 1913, donated by Count Michał Czarnecki (a local landowner at the time). Unfortunately, we have no further information about the immediate context of its discovery apart from the fact that it was found in a burial ground of the Lusatian culture. The cemetery itself was already known from earlier research that was conducted on it in 1891 by Bolesław Erzepki.

The assemblage of the hoard from Przybysław included a socketed bronze axe, a fragment of a necklace with a twisted shaft, two fragments of a necklace that was made of two rods that were cast together, three fragments of two elements from a collar breastplate, and three slabs (ingots) of raw materials (that were described so far as one slab of bronze) and two wedge-shaped slabs of iron raw material [48]. On the basis of the typological analysis, the chronology of this assemblage was set during the HaD period (Hallstatt D) (i.e., around 600 BC) [49,50]. It was noted that, during this period, the supply of bronze was reduced, so more of the material needed to be recycled in order to meet the demand for everyday artefacts [51].

Non-processed raw metal from the Bronze Age and Early Iron Age is exceptionally rare in Poland; it is more common to find pre-prepared raw materials that were shaped as bars or ingots. Some types of products from among the jewellery and axes are also interpreted as raw materials (or as a specific form of pre-monetary character). According to a compilation of the hoards that date backing to the HaD period from the area of present-day Poland, the frequency of the occurrence of raw materials in their composition is 6% [49].

The exceptional nature of the analysed hoard from Przybysław is also evidenced by the fact that it belongs to the bimetallic treasures. The iron raw material from it was analysed by Jerzy Piaskowski [52]; his results indicated that it was bloomery iron that contained a large amount of contaminants, which may indicate the local origin of the iron raw material.

Although very important for the analysis of those technological issues that are related to the production of bronze, the purpose and use of the finished products, and the interpretation of the symbolism that is associated with them, the type of category of buried hoard often poses many problems due to the states of the preservation of the analysed objects. This is because it is necessary to distinguish primary damage from that which has been caused by post-depositional processes [49].

## 2. Materials and Methods

**Figure 1:**
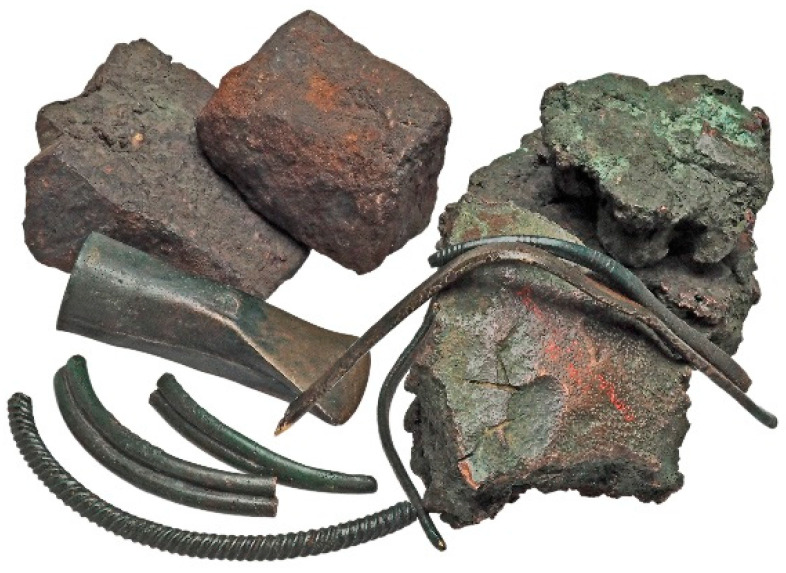
Hoard found in Przybysław including three lumps of raw bronze material, two lumps of iron raw material, six fragments of four necklaces, and a socketed axe.

Discovered in Przybysław in Greater Poland in a necropolis that was associated with a Lusatian Culture community, the hoard (Figure 1) was analysed and dated to c. 600 BC HaD (Hallstatt D). Due to the proportion of the ingots of raw materials and the damaged products for remelting in the find, the hoard was attributed to metallurgical activity. Therefore, we can study it as a metallurgist’s hoard (which makes it a unique find in the territory of the Polish lands) or analyse it in the context of its diversity (which allows us to show both the materials that were used and the manufacturing techniques).

Deposit studies were carried out on the three fragments of the raw copper material (Nos. AGH Prz.184i: R1, R2, R3), a bronze axe (No. AGH Prz.184a), two necklace fragments (No. AGH Prz.184b), fragment of a necklace with a twisted shaft (No. AGH Prz.184c) and three fragments from two parts of collar breastplates (Nos. AGH Prz.184d, e, f).

The aim of the study was to characterise the assemblage from metallographic and technological points of view. Macroscopic and microscopic examinations were carried out using stereo and metallographic microscopes. The chemical composition was determined by energy dispersive X-ray fluorescence spectrometry (ED-XRF). The surface topography and microstructure were analysed using a scanning electron microscope with an energy dispersive X-ray spectroscopy (EDS) system and electron backscatter diffraction detector (EBSD).

A thermodynamic analysis of the selected multi-phase alloys was carried out using the CALPHAD (CALculation of PHAse Diagrams) system [53,54]. Together with a phase diagram analysis [55,56], this method allowed for the phase transformations and phase compositions of the studied alloys to be identified. The results of the study were correlated with an experiment in which model (reference) alloys that were identical to the original alloys in their composition were prepared and cast in ceramic, sand, and metal moulds. The experimental (model) alloys were tested for tensile strength, ductility, and hardness.

### 2.1. Macro- and Microstructure Analyses

Macroscopic surface observations were carried out using a NIKON SMZ 745T stereo microscope. Metallographic microstructural observations were made using an optical microscope (NIKON EclipseLV150, Tokyo, Japan) with a camera, Nis-Elements image registration, and analysis software (Melville, NY, USA) Ver. 3.22.15 (Build 738).

SEM-EDS analyses were performed using a Hitachi S-3400N scanning electron microscope (Tokyo, Japan) with Thermo Noran energy dispersive X-ray spectroscopy (EDS) and a TESCAN MIRA high-resolution microscope (Brno, Czech Republic) that was equipped with an FEG Schottky electron emission source (SW-Version 1.1.3.0, Build 4348) with a high-powered tungsten gun as an electron source.

### 2.2. Chemical Composition Analyses and Phase Analysis

The chemical composition was determined by energy-dispersive X-ray fluorescence spectrometry (ED-XRF) using a Spectro Midex spectrometer (manufactured by SPECTRO Analytical Instruments GmbH, Kleve, Germany) that was equipped with a Mo anode X-ray tube and an Si SDD semiconductor detector. The composition of the modelling alloys was verified using a SPECTROMAXx emission spectrometer (SPECTRO Analytical Instruments GmbH, Kleve, Germany) with z iCAL 2.0.

A micro-area chemical analysis was performed by energy dispersive X-ray spectroscopy (SEM-EDS) using an Oxford Instruments SDD Ultim Max EDS detector (manufactured by Oxford Instruments, Abingdon, UK). Microstructural analyses were also performed using an electron backscatter diffraction detector (EBSD) by Symmetry S2 (manufactured by Oxford Instruments), using AZtecHKL data acquisition software and complemented by the AZtecCrystal data processing platform. To determine the mineral/phase and chemical composition of the ingot, structural X-ray diffraction (powder X-ray diffraction or PXRD) was employed using a Rigaku SmartLab diffractometer (Rigaku, Tokyo, Japan). This technique enabled the identification of the mineral and phase composition, relying on a microsample extracted from the ingot.

### 2.3. Thermal-Derivative Analysis (TDA) and CALPHAD Phase Transition Modelling Method

Thermal derivative analysis (TDA) was used to record and determine the characteristic thermal effects that resulted from the phase transformations that occurred during the crystallisations of the studied CuSn and CuPbSn alloys.

Derivative thermo-analysis (ATD), the method of analyzing differential curves of the cooling process, is a tool for obtaining information about the crystallization of individual phases formed during the crystallization of the alloy. Using the derivative curves, we can clearly identify a number of characteristic points of change. Crystallization of the sample took place in an isolated mold to achieve a low cooling rate. Temperature measurements were made using a Keysight 34972A laboratory multimeter (Santa Rosa, CA, USA) that was equipped with a 16-channel Reed Module 34902A multiplexer. The data that were obtained allowed for thermal analyses of the tested CuSn and CuPbSn alloys.

Phase analysis studies were also carried out based on TDA curves and thermodynamic modelling. The CALPHAD (CALculation of PHAse Diagrams) method, i.e., a method for calculating phase diagrams, was used to model the thermodynamic parameters of phases. This method is also very useful for simulating the behavior of complex multiphase systems. The idea of the CALPHAD method calculation is based on the calculation of the Gibbs energy of the phases analysed (as a function of temperature and as a function of their chemical composition) [53]. The CALPHAD method shows the relationship between the content of elements in the system and the equilibrium amounts of stable phases at a given temperature, which is the first step to being able to calculate the properties of the considered material [54,55,56]. The thermodynamic analysis of the alloys was carried out using the CALPHAD method with the Thermo-Calc Ver. 3.1 package for the TCS Cu-based Alloys Database (TCCU) of copper alloys.

### 2.4. Preparation of Model Alloys

The aim of the study was to characterise selected alloys from the Przybysław deposit by means of the developed method of model alloys (standards). This method allowed for evaluations of the structures and properties of the alloys that were used in the Bronze Age (which were different from those of today). It also allows for the characterisations of their cooling and crystallisations without interfering with the monumental material (i.e., in a non-destructive manner) through their experimental reproduction and theoretical and experimental analyses of the models of these alloys.

The alloy-melting process was carried out in a medium-frequency electric induction furnace using a chamotte-graphite crucible. The primary feedstock consisted of technically pure metals: copper, lead, tin, antimony, iron, zinc, bismuth, nickel, and silver. Arsenic was introduced into the alloy using a CuAs30 mordant. Individual alloying additives were introduced into the molten copper at 1150 °C. To protect against oxidation and hydrogen absorption, the copper was melted under a charcoal cover. Recordings of the cooling curves of the tested alloys were made at a pouring temperature of 1200 °C. Samples for the remaining tests were cast from a lower temperature of 1050 °C. The selected alloys were cast for the strength tests into metal, ceramic, and sand moulds. The alloys were tested in order to determine their chemical compositions, HBS hardnesses, UTS tensile strengths, A elongations, and microstructures.

### 2.5. Analysis of Alloy Properties

The study of the mechanical properties of metals and alloys includes, among other things, the determination of the UTS tensile strength, elongation A, and hardness, which are the basic service parameters of materials. The UTS tensile strength and elongation A were determined using Labor Tech’s LabTest ZD20 (V = 10 MPa/s), while Brinell hardness measurements of HBS 2.5/62.5 were taken using an HPO-250 hardness tester (WPM, Labor Tech, Leipzig, Germany).

The determination of the mechanical properties of the analysed alloys was carried out in accordance with the applicable standards, thus ensuring the accuracy of the measurements. Mechanical property tests were carried out in accordance with ISO 6892-1 [57]. Hardness measurements were carried out in accordance with EN ISO 6506-1 [58].

## 3. Results

### 3.1. Macro- and Microstructure Analyses

Selected results of macroscopic and microscopic analyses are presented in Figure 2, Figure 3, Figure 4, Figure 5, Figure 6, Figure 7, Figure 8, Figure 9, Figure 10, Figure 11, Figure 12, Figure 13, Figure 14, Figure 15, Figure 16, Figure 17, Figure 18 and Figure 19. The metallographic tests were carried out on the prepared samples in order to assess any changes in the microstructures (taking the individual precipitations into account).

**Figure 2:**
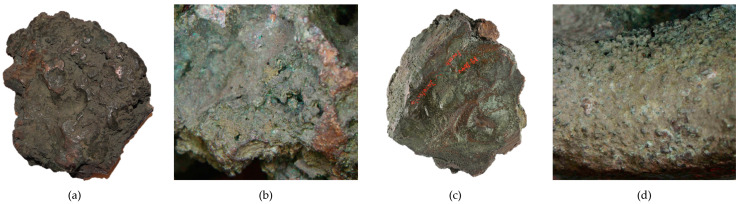
Macrostructure of the Prz.184i_R1 ingots: top of ingot (**a**); an apparent volumetric shrinkage effect closer to centre of slab (**b**); bottom of ingot (**c**); fragment with visible rim (**d**).

**Figure 3:**
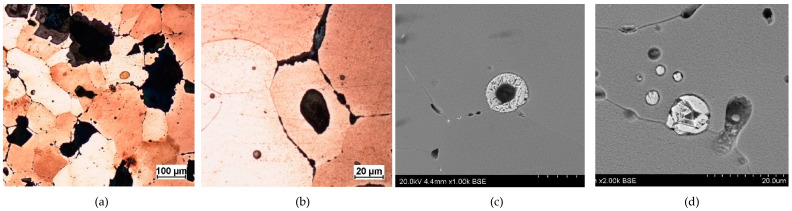
Microstructure of the Prz.184i.R1 ingot: 100× (**a**); 500× (**b**); 1000× (**c**); 2000× (**d**).

**Figure 4:**
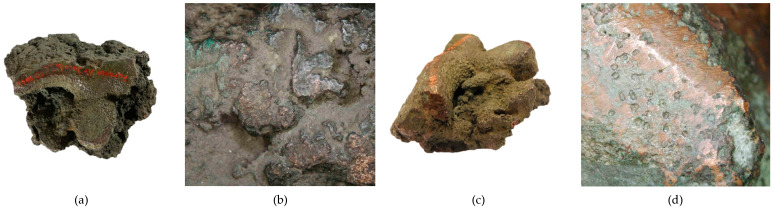
Macrostructure of the Prz.184i_R2 ingot: top of ingot (**a**–**c**); an apparent volumetric shrinkage effect (**b**); bottom of ingot (**d**).

**Figure 5:**
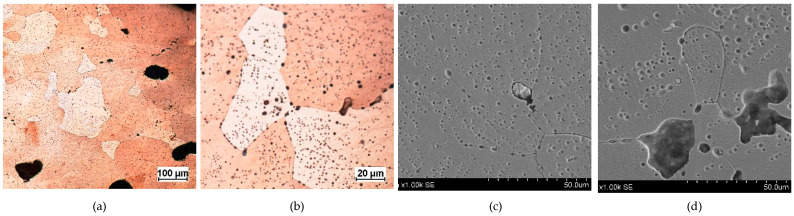
Microstructure of the Prz.184i.R2 ingot: 100× (**a**); 500× (**b**); 1000× (**c**,**d**).

**Figure 6:**
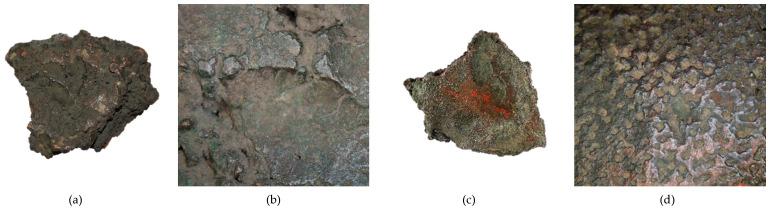
Macrostructure of the Prz.184i_R3 ingot: top of ingot (**a**,**b**); bottom of ingot (**c**,**d**).

**Figure 7:**
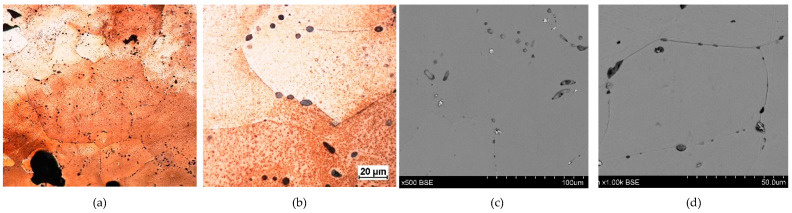
Microstructure of the Prz.184i.R3 ingot: 100× (**a**); 500× (**b**,**c**); 1000× (**d**).

**Figure 8:**
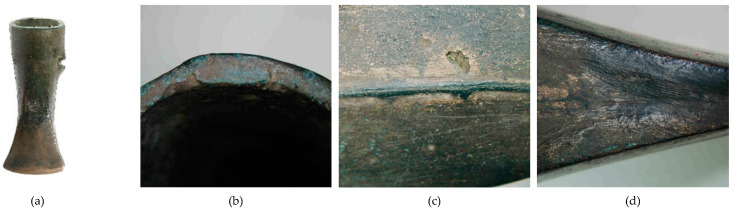
Macrostructure of the Prz.184a axles: general view (**a**); appearance of the gating system (**b**); fin (burr) on the parting plane (**c**), shrinkage depression (**d**).

**Figure 9:**
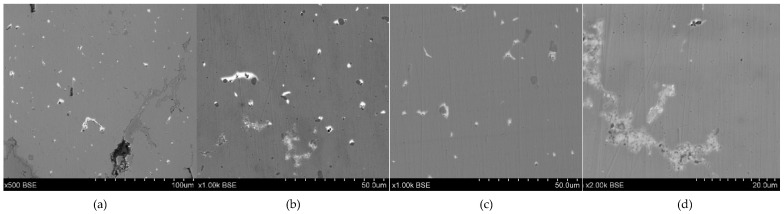
Microstructure of the Prz.184a axes: 500× (**a**); 1000× (**b**); 1000× (**c**); 2000× (**d**).

**Figure 10:**
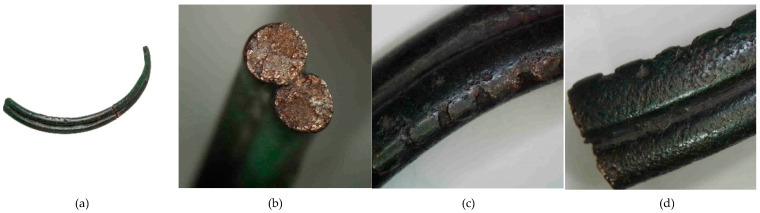
Macrostructure of the Prz.184b necklace: general view (**a**); breakthrough (**b**); ornamentation (**c**,**d**).

**Figure 11:**
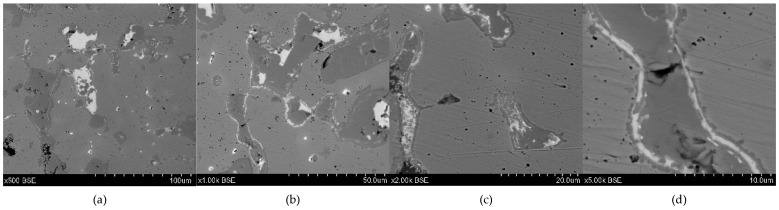
Microstructure of the Prz.184b necklace: 500× (**a**); 1000× (**b**); 2000× (**c**); 5000× (**d**).

**Figure 12:**
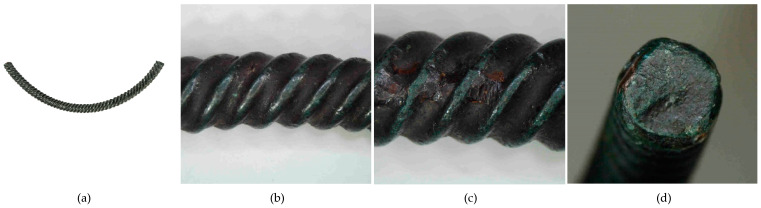
Macrostructure of the Prz.184c necklace: general view (**a**); tordering (**b**,**c**); breakthrough (**d**).

**Figure 13:**
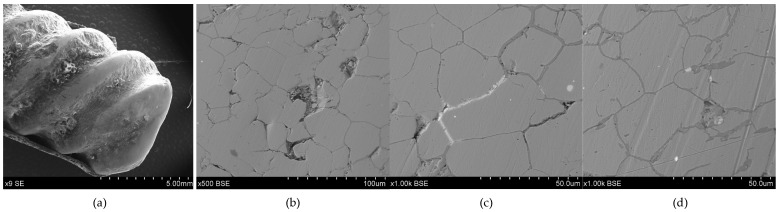
Microstructure of the Prz.184c necklace: 9× (**a**); 500× (**b**); 1000× (**c**); 1000× (**d**).

**Figure 14:**
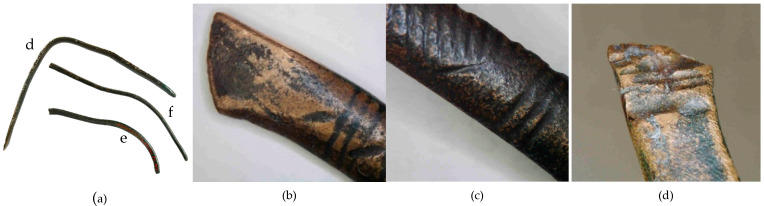
Macrostructure of the Prz.184d necklace: general view of necklace fragments Prz.184d-f (**a**); ending of necklace (**b**); ornamentation (**c**); traces made with a chisel for the secondary division of necklace (**d**).

**Figure 15:**
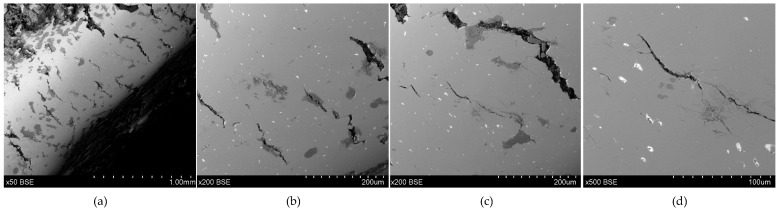
Microstructure of the Prz.184d necklace: 50× (**a**); 200× (**b**); 200× (**c**); 500× (**d**).

**Figure 16:**
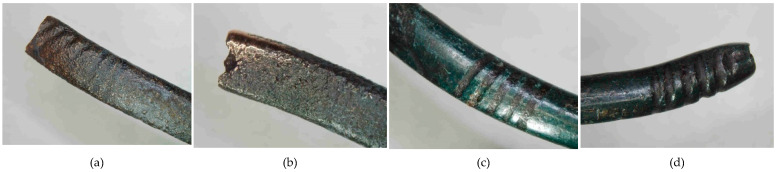
Macrostructure of the Prz.184e necklace: breakthrough (**a**,**b**); ornamentation (**c**,**d**).

**Figure 17:**
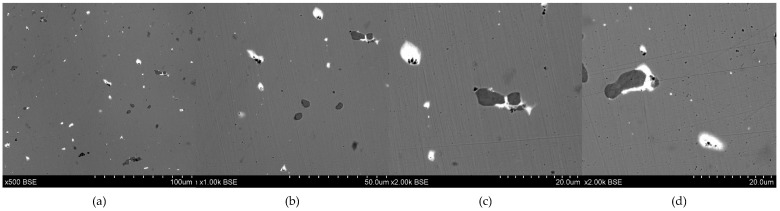
Microstructure of the Prz.184e necklace: 500× (**a**); 1000× (**b**); 2000× (**c**); 2000× (**d**).

**Figure 18:**
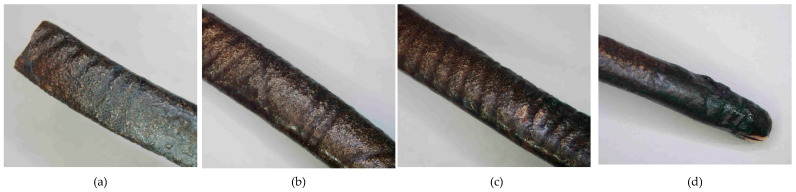
Macrostructure of the Prz.184f necklace: breakthrough (**a**); ornamentation (**b**–**d**).

**Figure 19:**
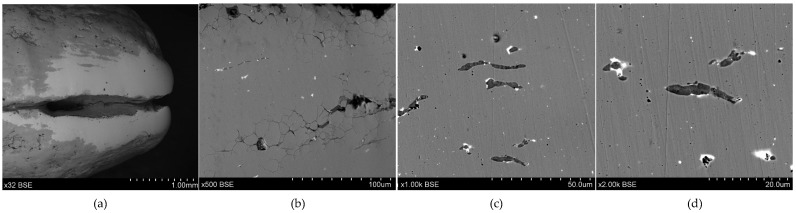
Microstructure of the Prz.184f necklace: 32× (**a**); 500× (**b**); 1000× (**c**); 2000× (**d**).

The following elements of the deposit were examined:

- Fragment of raw material R1 (Prz.184i; Figure 2 and Figure 3): A broken fragment of a larger piece of an almost circular shape with a diameter of 25–30 cm (known as an ingot) and cast in what was probably an open sand mould. The photograph shows a surviving fragment with the visible edge of the slab with raised edges and an apparent volumetric shrinkage effect closer to the centre of the ingot. The walls of the mould must have been undercut to facilitate the removal of the mould. The upper surface of the disc shows traces of after-machining, perhaps to level the surface and offset the effect of the free solidification surface. The underside of the ingot also shows traces of after-treatment. The microstructure shows numerous porosities (Figure 3a,b) and small metallic inclusions in the copper matrix (Figure 3c,d).

- Fragment of raw material R2 (Prz.184i; Figure 4 and Figure 5): As before, this was smelted from metal ore and cast in the form of a circular slab of about 25 cm in diameter, then broken to form a fragment from a larger whole. The fracture and fragment of the collar are clearly visible. Numerous casting defects in the forms of porosity and shrinkage cavities are visible on the surface. A tool mark (chisel) is visible in the centre, which may indicate the intention to break (division) it further into smaller fragments. Both the R1 and R2 fragments may have come from the same slabs. The microstructure shows extensive porosity, and numerous small gas inclusions (Figure 5a,b,d), and small metallic precipitates in the copper matrix (Figure 5c).

- Fragment of raw material R3 (Prz.184i; Figure 6 and Figure 7): As before, this is a piece that was broken off from a larger whole. There are clearly visible hammer marks on both sides, as well as traces of attempted chiselling or deliberate process marks. The fracture shows the direction of the crystallisation from bottom to top. The microstructure shows porosity and Cu-Cu_2_O oxygen eutectics at the grain boundaries. There are also finely dispersed metallic inclusions at the grain boundaries, indicating the presence of lead (Figure 7b,d).

- Socketed axe (Prz.184a; Figure 8 and Figure 9): This was cast in a two-piece mould, with the core forming the inner part of the casting. The metal was poured into the mould cavity through a runner cup that branched into two running gates (the remains of which can be seen on the axe flange). A flash is visible in the plane of the hatchet split, indicating the leakage or wear of the mould. Shrinkage dimples are visible on both sides of the axe, resulting from volumetric shrinkage and a decrease in the pressure of the liquid phase within the casting. Small gas porosities are visible on the surface and in the centre of the axe—defects that resulted from the reaction of the liquid metal to the mould and core material. The surface of the axe shows traces of the finishing and sharpening of the blade. The mechanical failure of the ear was caused by the use of the axe; an indirect cause of this may have been an internal defect in the casting. The microstructure of the axe shows two types of precipitates: white (which indicates the presence of lead), and grey (which is the residue of the sulphides that were extracted from the copper ore) (Figure 9).

- Two fragments that form one half of a broken necklace, made by the casting technique (Prz.184b; Figure 10 and Figure 11) [23]: The necklace consists of two round rods that were joined together during the casting; these taper and join at the ends. In addition, the necklace is decorated with six successive notches that occur in four sequences near the end of the necklace (Prz.184b). These decorations were made on a wax model, and then their negatives were mapped in a clay mould. The investigations and simulations that were carried out allowed the causes of the defects in the necklace to be determined experimentally [23]. The microstructure of the necklace shows numerous intermetallic phases (Figure 11).

- Fragment of a necklace with a twisted shaft, known as a torque or a neck ring (Prz.184c; Figure 12 and Figure 13): This forms one-third of a regular circle that had an external diameter of 153 mm. A break is visible on both sides, indicating mechanical damage. The cross-section of the necklace is similar to a circle on one side and a square on the other. The necklace is decorated with parallel, regular tordering along its entire length. It is not known whether the tordering was produced by plastic working via twisting the shaft around an axis, or by making the decoration on a wax model and casting the finished pattern (so-called pseudo-tordering). A breakthrough of the necklace with a characteristic defect that indicates the plastic deformation of the material may be decisive (Figure 12d). The microstructure of the necklace shows the effects of plastic processing and heat treatment (Figure 13b,d).

- Fragment of a necklace with a rectangular cross-section in the form of a flattened shaft (Prz.184d, Figure 14, Figure 15): One end is convex and polished, flattened from the top, and decorated with vertical and oblique incisions. The other flattened end shows a break as well as traces of incisions that were made with a chisel for the secondary division of the necklace (for recycling and to obtain raw material or intentional destruction). The microstructure shows numerous cracks that were caused by the bending of the necklace element (Figure 15). The necklace fragment may have been a set with two other pieces Prz.184e and f, (Figure 14a, Figure 16, Figure 17, Figure 18 and Figure 19).

- Fragment of a necklace in the form of a flattened shaft with a rectangular section on one side and a slightly rounded oval section on the other (Prz.184e; Figure 14a, Figure 16 and Figure 17) A crack is visible, indicating damage to the necklace. The other end is reworked, curved at the top and sides, and finished flat. Ornaments in the forms of parallel and diagonal incisions are visible. The microstructure shows minor lead and sulphide precipitations (Figure 17).

- Fragment of a necklace (Prz.184f; Figure 14a, Figure 18 and Figure 19): This matches broken necklace fragment Prz.184e and has a similar shape and decoration. The elongated shape of the fractures and grain boundaries that are visible in the microstructure confirm the plastic and heat treatment that was carried out to form the shaft into a necklace piece (Figure 19).

### 3.2. Chemical Analysis

The chemical composition analyses of all of the objects that were carried out by energy dispersive X-ray fluorescence spectroscopy are shown in Table 1. The tests were carried out on exposed surfaces.

The analysed Prz.184i.R1-R3 raw materials had such a similar chemical composition that they can be considered to have come from the same type of material (and even from the same smelting). They contained natural components that were accompanying elements of the copper in the ores: lead (0.9–1.25%), bismuth (0.25–0.37%), arsenic (0.21–0.4%), zinc (0.14–0.18%), silver (0.11–0.15%), and others. A study by the authors [59,60] shows that even a small proportion of arsenic in copper, relative to pure copper, slightly lowers the melting point and increases the crystallisation range of the alloy. The properties of Cu and Cu-As in the cast state are comparable but change after plastic processing and heat treatment.

**Figure 20:**
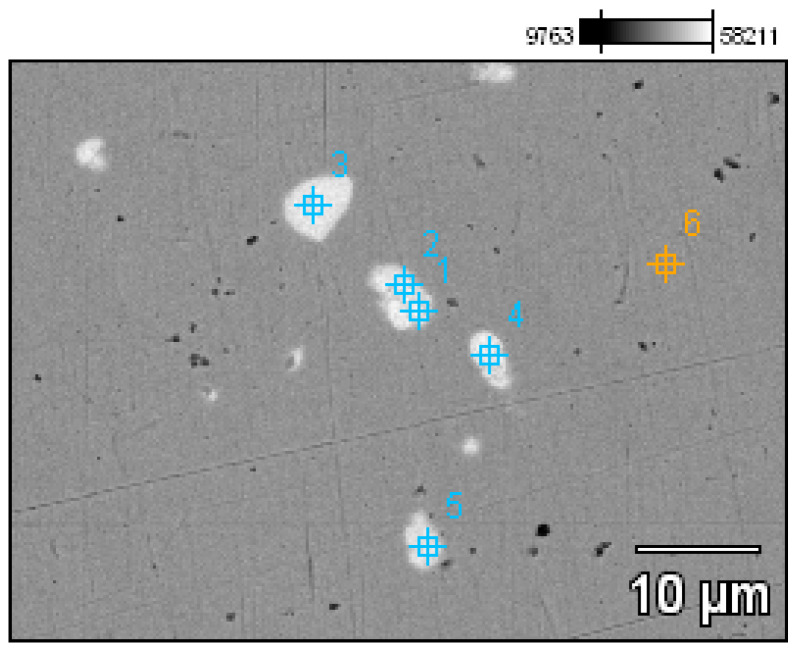
Microstructure of the Prz.184i.R2 ingot with the areas of chemical composition analysis in the micro-areas (Table 2).

**Figure 21:**
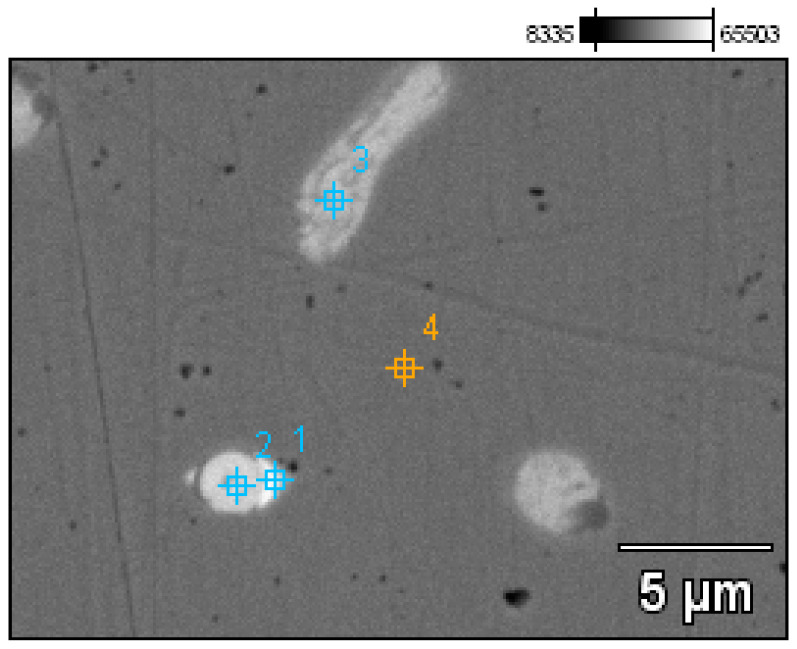
Microstructure of the Prz.184i.R2 ingot with the areas of chemical composition analysis in micro-areas (Table 3).

A detailed analysis of the microstructure was carried out using SEM-EDS (Figure 20). In the microstructure of the ingot, finely dispersed lead and arsenic precipitations could be observed against the copper matrix. The analyses that were carried out on these precipitations (points 1–5, Table 2, Figure 20) showed that the arsenic was within a range of 11.0–14.1%, and the lead was within 65.8–73.5%. In addition, antimony (1.5–2.1%), tin (2.4%), and bismuth (6.3%) were identified. The silver, arsenic, bismuth, and lead in the copper microstructure were confirmed in the area that is indicated in Figure 21 (Table 3).

**Figure 22:**
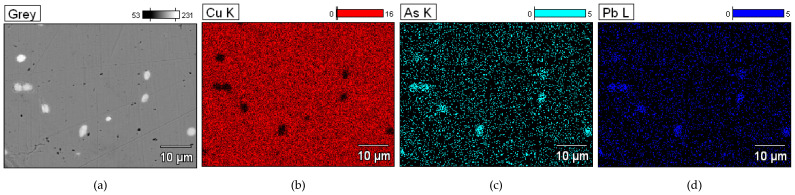
Microstructure of the ingot Prz.184i.R2. Map of elemental distribution: area of analysis (**a**), the presence of Cu (**b**); the presence of As (**c**); and the presence of Pb (**d**). Mag: 2000×.

The distribution of the elements is illustrated by a map of the occurrence of arsenic and lead in the copper matrix (Figure 22). It should be noted that the raw copper material was not alloyed with the tin, so it can be considered to have been a primary raw material, and the accompanying elements were ore-derived elements that remained in the structure during the smelting from the ore.

**Figure 23:**
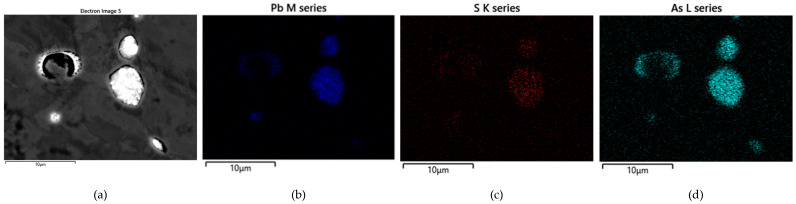
Microstructure of the Prz.184i.R3 ingot. Map of elemental distribution: area of analysis (**a**); the presence of Pb (**b**); the presence of S (**c**) and the presence of As (**d**) (mag: 10,000×).

**Figure 24:**
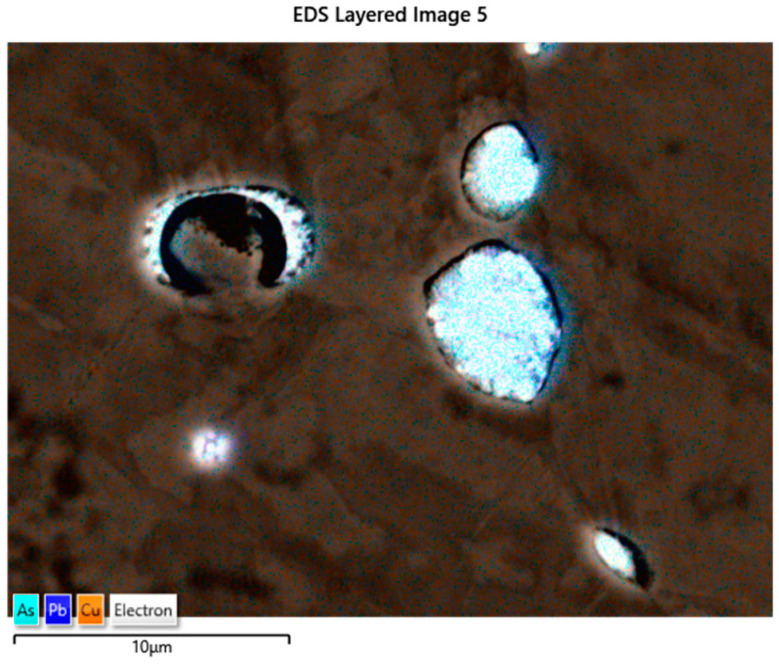
SEM-EDS layered image of the Prz.184i.R3 ingot (mag: 10,000×).

**Figure 25:**
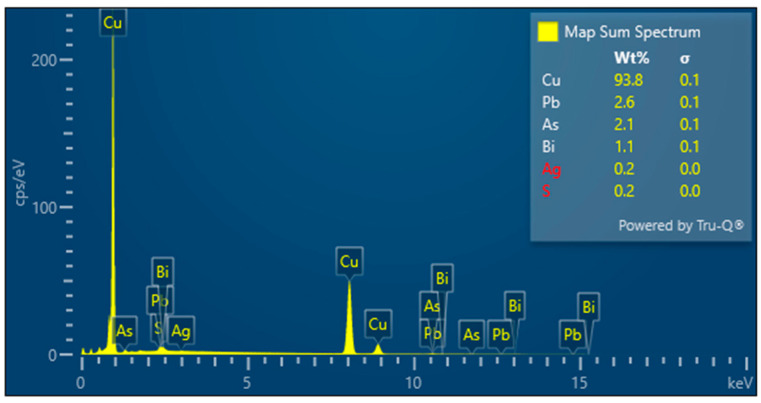
Elemental spectrum of the Prz.184i.R3 ingot and the quantitative elemental content in the micro-area of object (cf. Figure 24).

**Figure 26:**
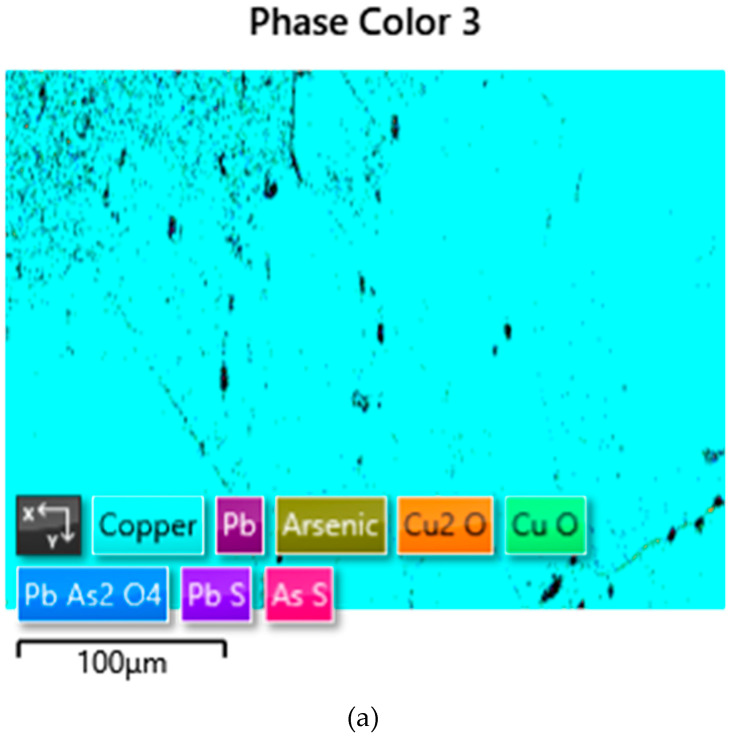

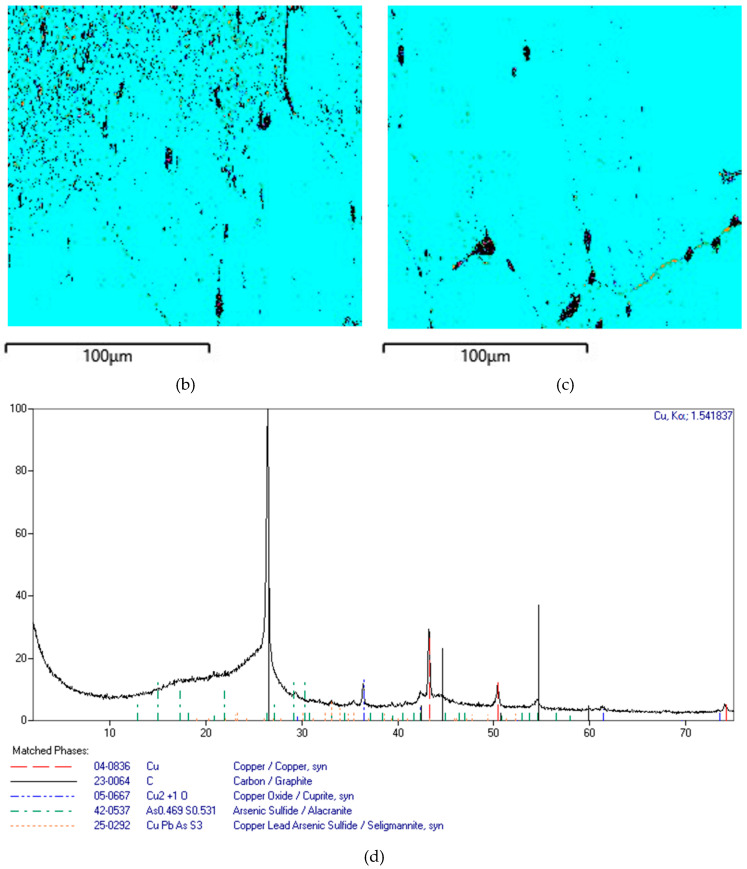
Phase-distribution map of the Prz.184i.R3 ingot (by EBSD) and phase identification (by PXRD): general view with phase identification (**a**); enlarged fragment—upper left area (**b**); enlarged fragment—lower right area (**c**); identification Cu, Cu_2_O, AsS, and CuPbAsS_3_ (**d**).

The EDS analyses of the Prz.184i.R3 ingot confirmed the presence of copper in the microstructure alongside Pb, As, Bi, Ag, and S (Figure 23, Figure 24 and Figure 25). Interpreted together, the EDS, EBSD, and PXRD analyses confirmed the presence of the following phases in the copper ingot: Cu, Pb, As, Cu_2_O, PbS, AsS, and CuPbAsS_3_ (Figure 23, Figure 24 and Figure 26; Table 4); the percentages of the phases are shown in Table 4. The matrix was copper. At the grain boundaries, lead was present in its pure form, as well as arsenic in the forms of PbS and AsS sulphides. Cu_2_S and copper sulphides were absent from the microstructure. Cu_2_O and the oxygen eutectic were also visible at the grain boundaries (Figure 26). The remaining oxides were the result of corrosion processes. On the basis of the PXRD, the presence in the copper raw material of copper and copper oxide—Cu_2_O, arsenic sulfide–AsS, and copper lead arsenic sulfide–CuPbAsS_3_ was confirmed (Figure 26d).

**Figure 27:**
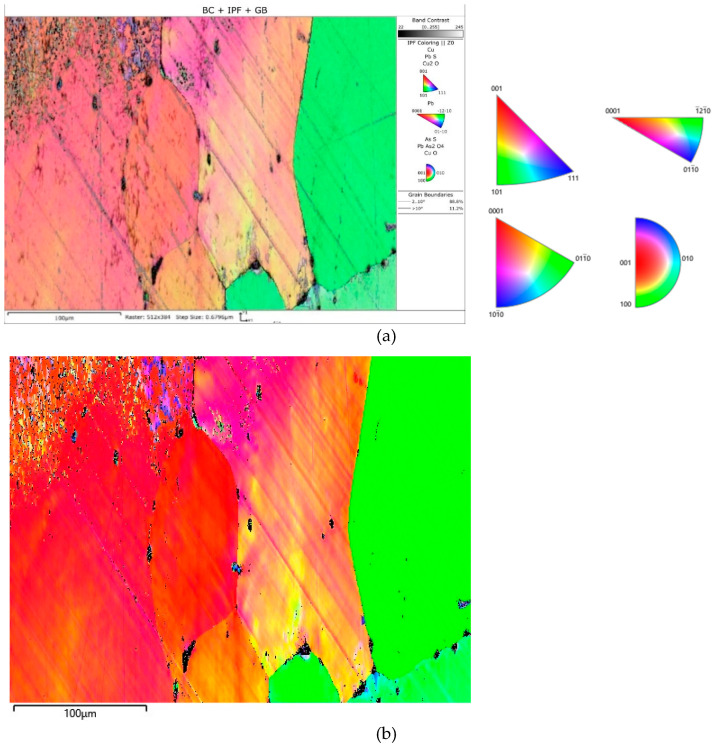
EBSD inverse pole figure (IPF) map of the Prz.184i.R3 copper ingot (**a**); IPF Z (**b**).

Figure 27 shows the crystallographic-orientation distribution of the listed phases (shown in IPF colours); the IPF colours were assigned according to the insets in the figure.

The EBSD analysis showed that the primary copper grains were large (Figure 27); this indicates a slow crystallisation process in a low heat-transfer form, (i.e., with slow heat removal from the solidifying ingot).

**Figure 28:**
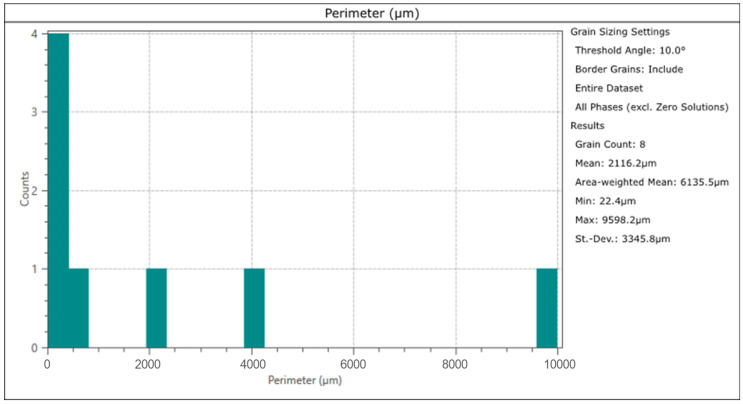
Copper grain size distribution in the Prz.184i. R3 ingot.

**Figure 29:**
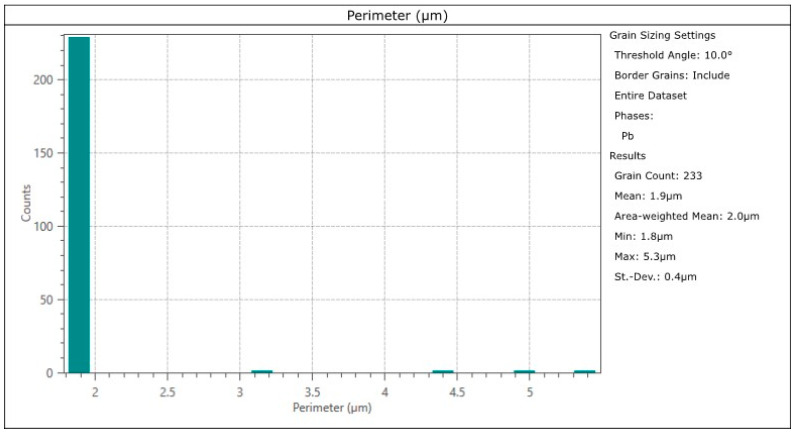
Circumferential size distribution of lead precipitations in the Prz.184i. R3 ingot.

The smallest copper grain circumference in the measurement area was 22.4 µm, and the largest was 9598.2 µm. The number of grains in this area was eight (Figure 27 and Figure 28). In comparison, the smallest lead grain circumference was 1.8 µm, and the largest was 5.3 µm. In the measurement area, 233 Pb grains could be identified (Figure 27 and Figure 29).

**Figure 30:**
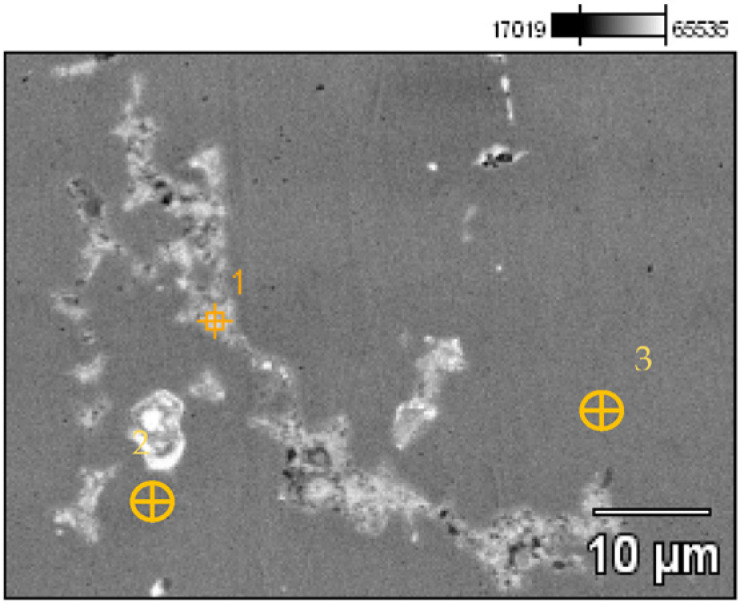
Microstructure of the Prz.184a axe, with the areas of chemical composition analysis in the micro-areas (Table 5).

**Figure 31:**
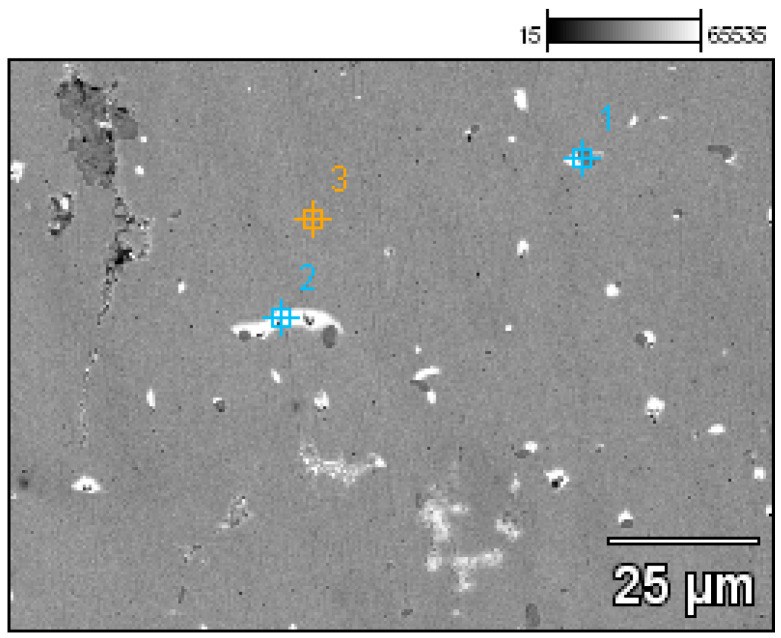
Microstructure of the Prz.184a axe, with the areas of chemical composition analysis in the micro-areas (Table 6).

Apart from the copper raw material from Przybysław, the other artefacts belonged to the group of tin-bronzes of the Cu-Sn or tin-lead Cu-Sn-Pb systems. For example, the bronze from which the axe was cast (Prz.184a) contained two alloy additions: 5.3% tin, and 1.9% lead (Table 1). It was therefore characterised by good casting and technological properties [59]. The microstructure shows an α (Cu) solid solution, a eutectoid, and areas with increased tin and lead contents (Figure 30 and Figure 31; Table 5 and Table 6). Of particular importance was the indication of the presence of minor copper(I) sulfide (Cu_2_S, Point 1, Figure 31)—in nature, it occurs as the mineral chalcogenide. Copper(I) sulfide was identified on the basis of atomic participation of elements in EDS analysis: 61.6 at% Cu and 38.4 at%. This indicated that the origin of the raw material came from sulphide ores and that the metallurgical process was not carried out fully (all of the undesirable components were not removed during the roasting).

**Figure 32:**
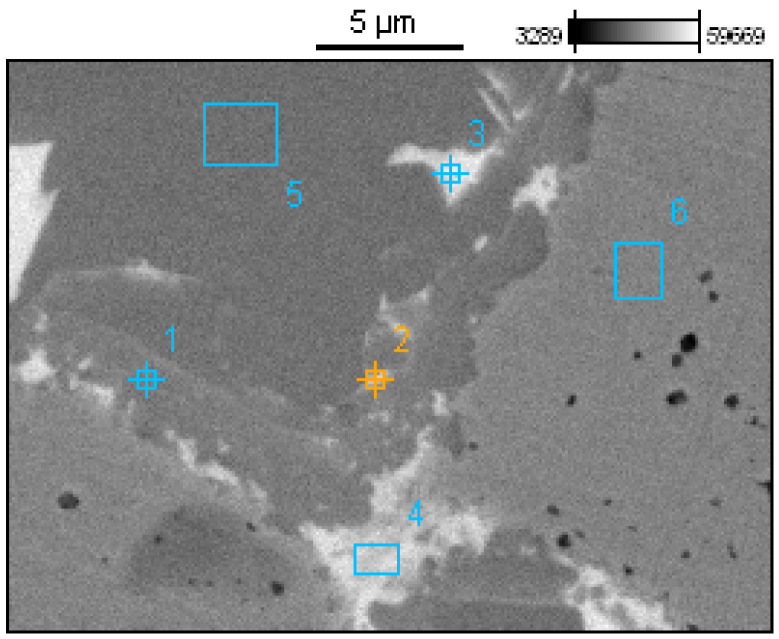
Microstructure of the Prz.184b necklace, with the areas of chemical composition analysis in the micro areas (Table 7).

The Prz184b necklace was made of lead-tin bronze, as was indicated by the lead content (6.4%) exceeding the tin content (2.4%) [35]. The elements that were associated with the origin of the copper ore were also identified: Bi, As, Sb, Ag, Ni, and Zn. It should be noted that lead bronzes have poorer mechanical properties than tin bronzes [60]. The chemical composition analyses revealed the presence of an α (Cu) solid solution and an eutectoid [35]. The presence of numerous lead-containing intermetallic phases was noteworthy (Figure 32, Table 7).

**Figure 33:**
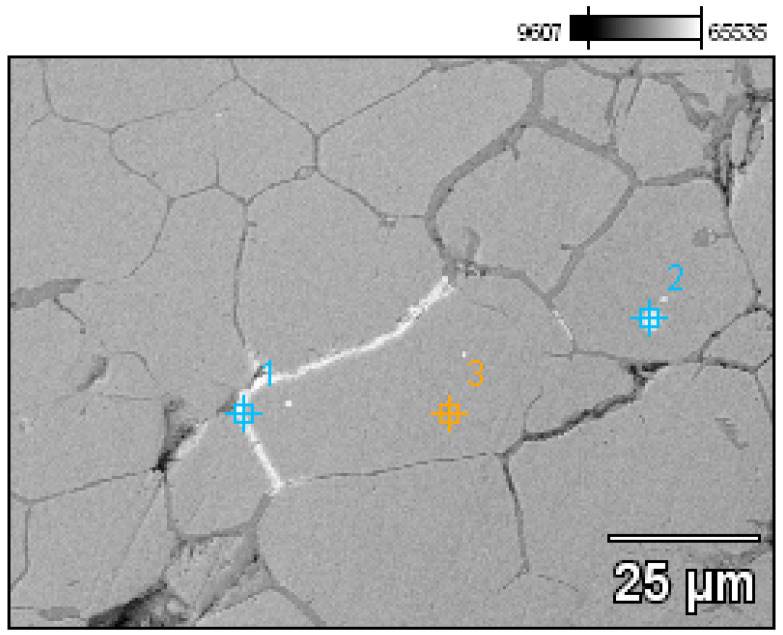
Microstructure of the Prz.184c necklace, with the areas of chemical composition analysis in the micro-areas (Table 8).

**Figure 34:**
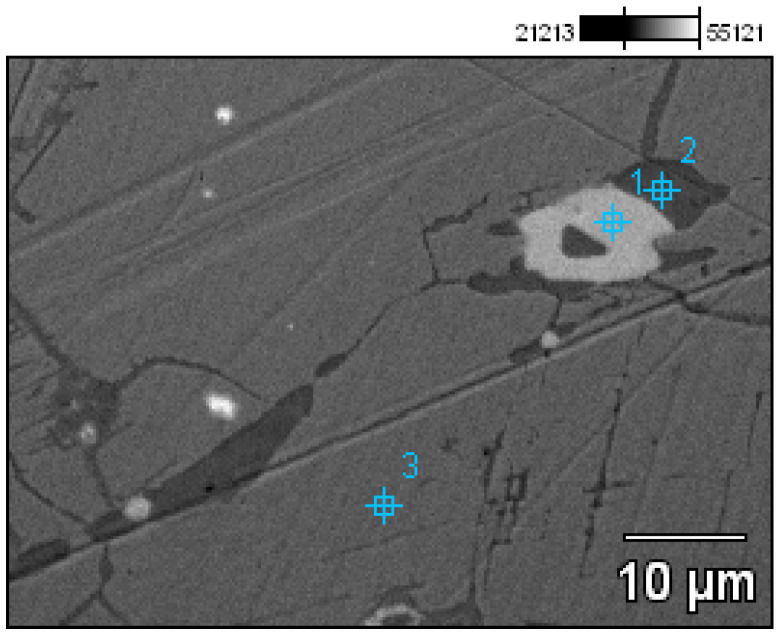
Microstructure of the Prz.184c necklace, with the areas of chemical composition analysis in the micro-areas (Table 9).

The most interesting artefact in the deposit was a fragment of a twisted-shaft necklace (Prz.184c). An analysis of its chemical composition indicated that it was cast from tin bronze, with a tin content of 8.5% (Table 1). The slight addition of lead (0.6%) probably indicates that the raw material came from the recycling of products that contained lead. Natural additives such as arsenic (0.3%), zinc (0.15%), nickel (0.12%), and iron (0.1%) were also important for the properties of the products. The lead in the microstructure was located both within the grains and at the grain boundaries (in the forms of prominent white areas) (Point 1, Figure 33, Table 8; and Points 1–2, Figure 34, Table 9). A detailed observation of the microstructure with analysis also revealed the presence of copper sulphides (Figure 34, Table 9). The corrosion, the visible intergranularity and the arrangement of the sulphides at the grain boundaries indicated that plastic and heat treatment took place.

**Figure 35:**
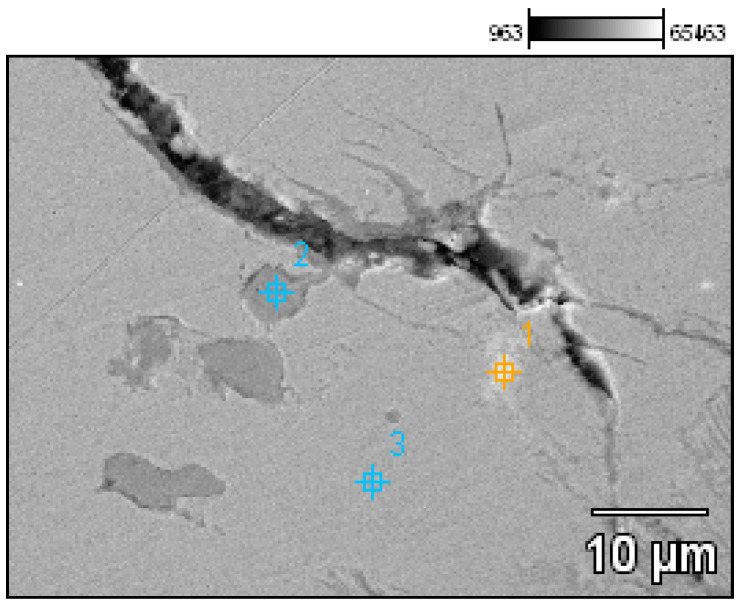
Microstructure of the Prz.184d necklace, with the areas of chemical composition analysis in the micro-areas (Table 10).

**Figure 36:**
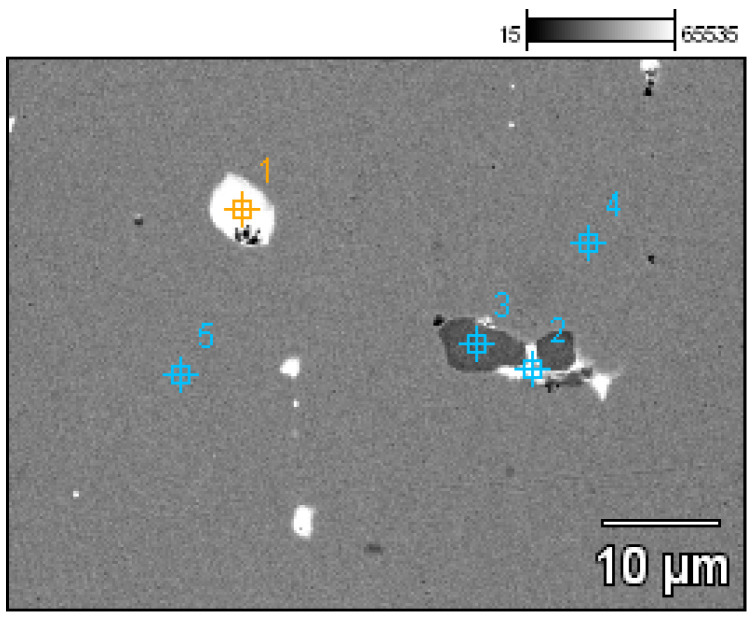
Microstructure of the Prz.184e necklace, with the areas of chemical composition analysis in the micro-areas (Table 11).

**Figure 37:**
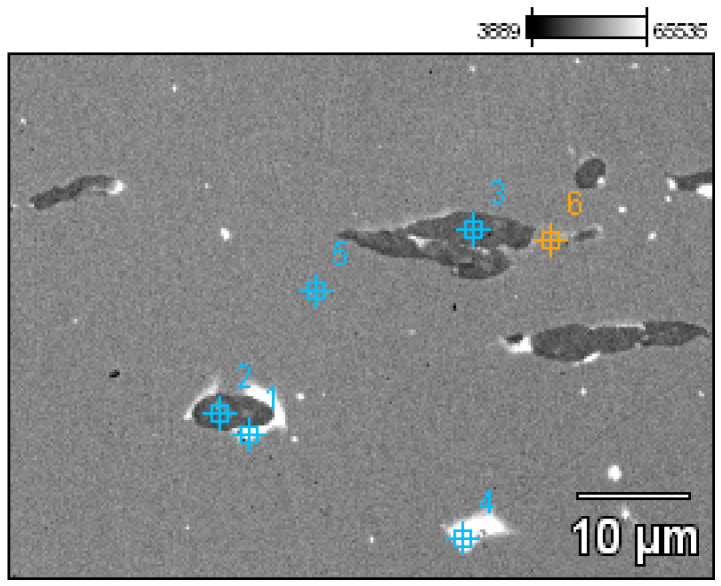
Microstructure of the Prz.184e necklace, with the areas of chemical composition analysis in the micro-areas (Table 12).

The chemical composition of the fragments of the Prz.184df necklace showed considerable similarities to tin bronze with lead. It was noteworthy that Prz.184d and Prz.184e were virtually identical, thus confirming that they belonged to the single ingot from which the necklace piece was made (8.2–8.4% tin and 0.9% lead); however, the piece marked “Prz.184f” contained slightly higher levels of tin (8.7%) and lead (1.4%) than the other. In both cases, the tin was an intentional addition, while the lead in such small amounts was the result of the remelting of the bronze raw materials that contained lead. As opposed to the microstructure of the Cu-Sn alloy, residual copper and iron sulphides were visible in the microstructures of Prz.184d and Prz.184e, along with lead precipitations (Figure 35 and Figure 36, Table 10 and Table 11). The similar nature of the microstructure and precipitates could also be found in the Prz.184f necklace (Figure 37, Table 12). However, the elongated shape of the sulphides was noticeable, indicating the plastic processing that moved place.

### 3.3. Preparation of Model Alloys

The purpose of the experiment was to determine the effect of alloy additives in the properties of the studied prehistoric alloys. Two characteristic alloys were selected from among the objects in the Przybysław hoard trove to serve as models. These were chosen on the basis of the production techniques that were used: the cast necklace (Prz.184b) and the twisted-shaft necklace with plastic processing (Prz.184c). The chemical compositions of the actual alloys are given in Table 1, while the chemical compositions of the model alloys in the successive casting stages are summarised in Table 13.

Corresponding to a cast necklace with the composition of CuPb6.5Sn2.4Bi0.8As0.3, the first alloy was produced in successive stages by adding lead (1), tin (2), and bismuth (3) as well as arsenic and other elements (4). Each time the chemical composition was analysed, a thermal analysis was carried out to see how the alloying elements affected the nature of the alloy crystallisation. Alloy 1 will be referred to as the CuPbSn alloy in the next stage of the experiment due to its lead content exceeding that of the tin content.

A model of the twisted-shaft necklace alloy with the composition of CuSn8Pb0.7As0.3Zn, the second alloy was produced by introducing all of the prepared components in a single process (1). Alloy 2 will be referred to as the CuSn alloy in the next stage.

The studies showed a concordance in the elemental proportions of the planned and realised melts (cf. Table 1 and Table 13).

### 3.4. Thermal-Derivative Analysis (TDA) Method and ThermoCalc Modelling

**Figure 38:**
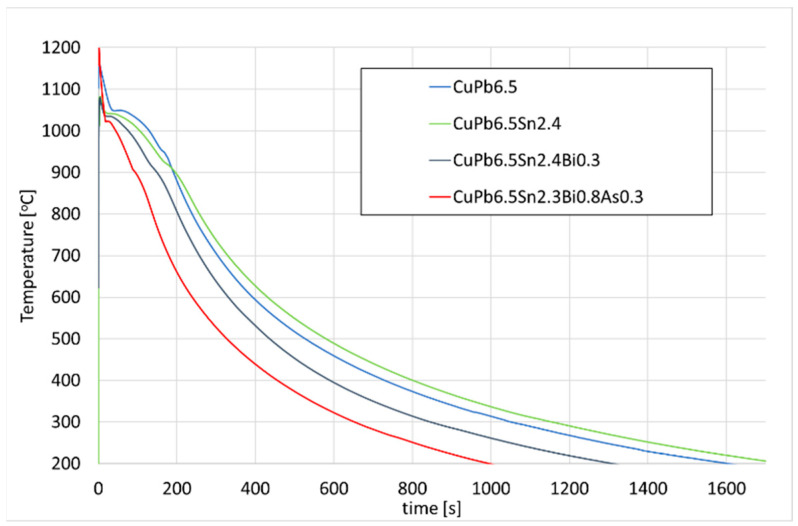
Curves that were recorded during crystallisation of the tested CuPbSn alloys with Sn, Bi, and As additives.

**Figure 39:**
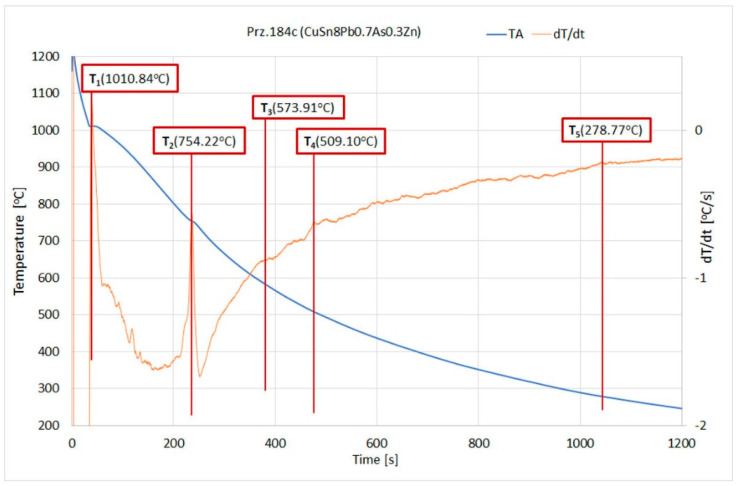
Phase-transition temperatures according to TDA during the CuSn alloy crystallisation (Prz.184c).

By recording the course of the crystallisation, it was possible to analyse the changes that occurred in the CuPbSn and CuSn alloys during their solidifications (Figure 38 and Figure 39). There was a noticeable reduction in the onset of the freezing point with each addition.

**Figure 40:**
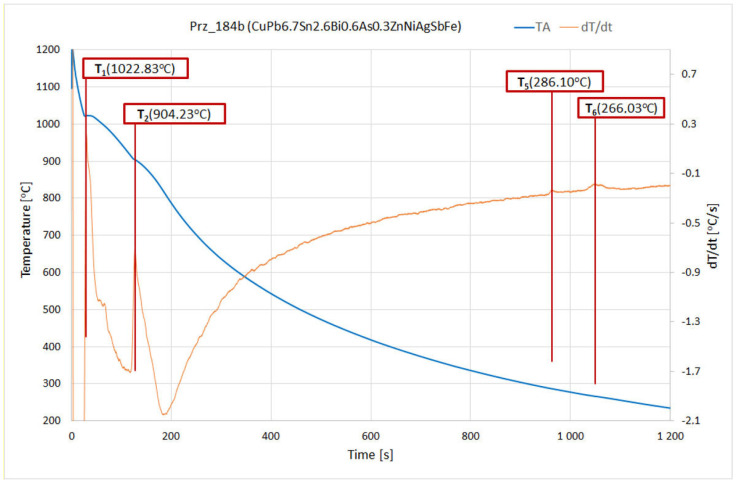
Crystallisation path of the CuPbSn system (Prz.184b) and phase-transition temperatures according to Thermo-Calc.

**Figure 41:**
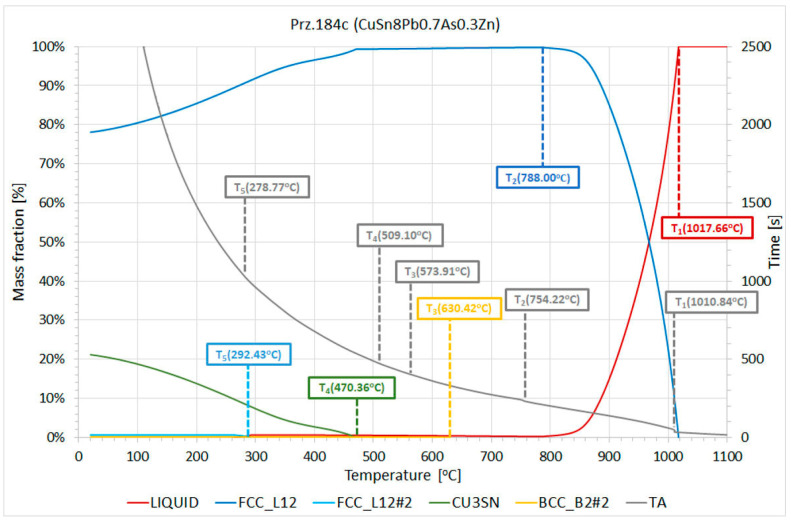
Crystallisation path of the CuSn system (Prz.184c) and the phase transition temperatures according to Thermo-Calc.

The modelling was carried out with the ThermoCalc program, which allowed the crystallisation processes of the studied alloys to be visualised (Figure 40 and Figure 41). The modelling results were compared with the actual processes that were recorded during the solidifications of the tested alloys and the thermal derivative analyses that were carried out in this manner. Table 14 shows the characteristic temperatures of the crystallisation processes of the selected alloys for the CuPbSn alloy casting experiment.

The theoretical (Thermo-Calc) and experimental (TDA) analyses (Table 14) showed that the crystallisation of the CuPb-type alloy was affected by the individual alloying additives. The TDA analysis showed that each successive element that was introduced (Sn, Bi, and As) decreased the crystallisation onset temperature T1 from 1048.9° to 1022.8 °C. Similar correlations also arose from the theoretical Thermo-Calc analysis; the T1 temperature slightly deviated in its values from the temperatures that were obtained by the TDA method, but the nature of the changes was similar. The slight differences in the temperatures of the TDA and TC analyses were mainly due to the unaccounted-for amounts of trace impurities that were present in the studied experimental alloys and the non-equilibrium conditions that occurred during the recordings of the crystallisation curves.

The solidification system of the CuPb6.5Sn2.3Bi0.8As0.3 alloy (Table 15) at T1 temperatures of 1038 °C (based on TC modelling) and 1022.8 °C (based on TDA analysis), involved the formation of the first crystals of an α(Cu) solid solution; this crystallised in an cubic crystallographic network, FCC_L12 (*face-centred cubic*), which was the basic structural component of the system in question. Upon analysing the TC modelling further, the next stable phases that were presented at an ambient temperature (20 °C) were BCC_B2 (*body-centred cubic*), CU3SN (*intermediate phases),* and FCC_L12#2 (*face-centred cubic*). The range of the occurrence of each phase is designated by its corresponding colour in Table 15. The BCC_B2 phase (which crystallised at T4 = 539 °C) was not recorded on the TDA crystallisation curve. The other phases (FCC_L12#2, CU3SN [crystallising at T5 = 293.6°/286.16 °C] and FCC_L12#3 [T6 = 270.18 °C]) were visible on the TDA curve.

The TC modelling took the non-persistent phases that were present in the respective temperature ranges into account during crystallization: BCC_B2#2 (within a range of 584.31°–539.94 °C), and CU10SN3 (408.55°–286.16 °C). The exact chemical compositions for the individual stable phases of the Prz.184b alloy (- CuPb6.5Sn2.3Bi0.8As0.3) that occurred at an ambient temperature were as follows:

-FCC_L12: 99.09% Cu, 0.39% Sn, 0.37% As, and 0.15% Zn;

-FCC_L12#2: 91.85% Pb, and 8.15% Bi;

-FCC_L12#3: 97.1% Ag, and 2.9% Sn;

-BCC_B2: 63.63% Co, 36.36% Fe, and 0.01% Bi; and

-Cu3Sn: 59.31% Cu, 38.44% Sn, and 2.25% Ni.

From the presented TC modelling, the range of the crystallisation of the alloy under study was 774.23 °C (1038.13°–263.9 °C); this was due to the presence of low-melting element in the alloy, such as lead and bismuth. However, taking the technological parameters (e.g., metal flowability and alloy fluidity) and, above all, the TDA analysis that was performed into account, the end of the actual crystallisation under non-equilibrium conditions was a recorded T2 temperature of 904.23 °C.

It should be noted that the presence of a low-melting element in an alloy (lead or tin) can cause the appearance of an unfavourable segregation phenomenon in special cases, especially the undesirable reverse segregation.

The crystallisation of the model alloy reflecting the chemical composition of Object Prz.184c (CuSn8Pb0.7As0.3Zn) that was performed by the TC modelling and the actual TDA analysis is graphically shown in Figure 41, and the characteristic recorded temperatures are summarised in Table 16. The T1 crystallisation onset temperature that was recorded by the TDA method was 1010.84 °C. Also recorded were the T2, T3, T4, and T5 temperatures, which were reflected in the theoretical modelling by the TC method. Temperature T2 (754.23 °C—TDA; 788.0 °C—TC) was practically at the end of the crystallisation; this was due to the decreasing amount of the liquid phase (0.0023%). Even small amounts of lead and bismuth in the alloy (low-melting elements, melting points: Pb = 231.9 °C and Bi = 271.4 °C) forced the appearance of the so-called “residual liquid” in Thermo-Calc modelling, the end of the crystallisation being determined by the T5 temperature (263.83 °C—TC). The range of the crystallisation of the model alloy in question was 753.8 °C for the TC modelling. The crystallisable stable phases that occurred at an ambient temperature during solidification were FCC_L12, FCC_L12#2, BCC_B2#2, and Cu3Sn, (the exact chemical compositions of which are shown below).

The chemical compositions for the individual permanent phases of the Prz.184c alloy (CuSn8Pb0.7As0.3Zn) that occurred at an ambient temperature were as follows:

-FCC_L12: 98% Cu, 0.47% Sn, 0.35% As, and 0.20% Zn;

-FCC_L12#2: 88.98% Pb, and 11.01% Bi;

-BCC_B2#2: 99.48% Fe, and 0.51% Ni;

-Cu3Sn: 61.06% Cu, 38.37% Sn, and 0.57% Bi.

The actual crystallisation range that was calculated from the TDA analysis is shown in Table 17.

### 3.5. Microstructure Analysis and Phase Analysis

**Figure 42:**
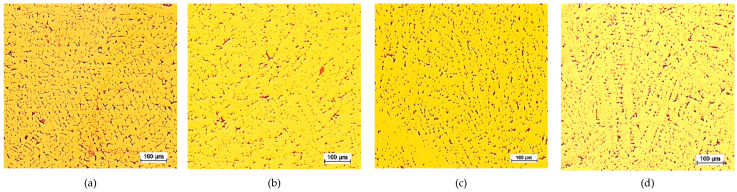
Casting mould, 100x: K1 CuPb (**a**); K2 CuPbP (**b**); K3 CuPbSn (**c**); K4 CuPbSnBi (**d**).

**Figure 43:**
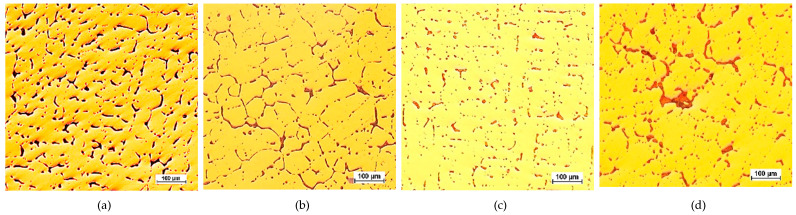
Sand mould, 100x: P1 CuPb (**a**); P2 CuPbP (**b**); P3 CuPbSn (**c**); P4 CuPbSnBi (**d**).

**Figure 44:**
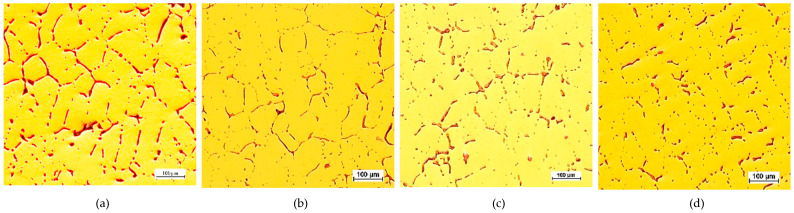
Ceramic moulds, 100x: C1 CuPb (**a**); C2 CuPbP (**b**); C3 CuPbSn (**c**); C4 CuPbSnBi (**d**).

The CuPbSn model alloy for the Prz.184b necklace was cast in sand, ceramic, and metal moulds in order to demonstrate the differences in the microstructures; selected results of these metallographic tests (OM) are shown in Figure 42, Figure 43 and Figure 44. The metallographic tests were carried out on the prepared specimens in order to assess any changes in the microstructures (taking the individual precipitations into account).

The microstructures of the CuPb alloy samples (Figure 42a, Figure 43a and Figure 44a) showed the dendrites of an α (Cu) solid solution and lead in the interdendritic spaces, in the forms of small round globules or larger elongated precipitations. After the addition of tin, the microstructures of the CuPbSn alloy samples showed solid α (Cu) solution dendrites and eutectoid (α + δ), as well as lead in the interdendritic spaces (Figure 42c, Figure 43c and Figure 44c). Following the introduction of further components, the microstructures did not change significantly, with some components entering the solutions and others forming intermetal phases in the interdendritic spaces (Figure 42d, Figure 43d and Figure 44d).

Comparing the microstructures of the castings from the metal, sand, and ceramic moulds, significant fragmentations of the structures were evident due to the rapid heat dissipation from the metal mould (Figure 42). The largest dendrites could be found in the ceramic mould (Figure 44), indicating the slowest heat dissipation from the mould cavity.

### 3.6. Analyses of Alloy Properties

The next stages of the research were to assess the changes in the properties of the CuPbSn alloy as a function of casting it into sand and metal moulds and compare them with the properties of the CuSn alloy. Strength tests were carried out on samples that were cast in machined metal moulds. Hardness measurements were carried out on samples cast in sand and metal moulds. A spiral flow test was also carried out on the analysed alloys. Metal runnability defines the ability to fill a mould with a liquid alloy through the a gating channel. The results of the study are summarised in Table 18.

The obtained strength-test results showed variable values of the analysed parameters for the CuPbSn (Prz.184b) and CuSn (Prz.184c) alloys. The CuPbSn (Prz.184b) alloy showed slightly lower UTS strength and HBS toughness as well as greater elongation. In contrast, the CuSn (Prz.184c) alloy showed higher strength and hardness as well as slightly lower elongation. The properties of the analysed alloys were mainly determined by the copper content and the interaction of the alloying elements that were present. For the CuPbSn alloy, the lead present in the structure was in the form of free precipitations, while the low tin content (2.3 wt.%) resulted in the solution strengthening of the copper. For the second alloy (i.e., CuSn), the main effect of the tin was due to its dissolution in the copper and the formation of eutectoid precipitations, which caused the alloy to strengthen in the UTS and HBS ranges. The difference in the hardness of the analysed model alloys that solidified in the metal and sand moulds indicated the sensitivity of these plastics in terms of the rates of the heat dissipation during the solidification and crystallisation processes. The spiral flowability test that was conducted showed the significantly better flowability of the CuSn alloy (Prz.184c); this was due to the content and interaction of the tin in the analysed bronzes.

## 4. Discussion

### 4.1. Analysis of Archaeological Material

Considering the fact that the Przybysław deposit contained fragmentary bronze objects and blocks of raw copper material, it can be technologically considered to be a deliberate deposit of raw materials that were may have been intended for re-melting in the foundry process.

The most important objects that were considered in the work were the three fragments of raw materials (Prz.184i.R1-R3) that were found in the forms of round ingot fragments. Observations of the ingot surfaces indicated open-mould casting. Numerous shrinkage porosities were visible, along with the effects of free-volume shrinkage near the centre of the ingot. The visible free-solidification surface indicated that the ingots were cast in open moulds. The ingots showed traces of forging on their surfaces, probably to consolidate the ingot and remove the surface defects. Traces of the use of a tool (a chisel) were also visible, which may have indicated the intention to further break the ingots into smaller fragments in order to prepare the melt. The microstructures of the ingots showed numerous small gas pores and small metallic inclusions in the copper matrix.

This raw material has, thus far, been mentioned in the literature as a raw bronze material. The ED-XRF studies unequivocally proved that it was a copper raw material that contained the natural additives that are commonly included in copper ores: lead, arsenic, bismuth, zinc, silver, and iron. In addition, the SEM-EDS and PXRD analyses showed the presence of lead precipitations and lead and arsenic sulphides in the microstructure, as was confirmed by EBSD. Cu-Cu_2_O oxygen eutectics were also visible at the grain boundaries. As indicated, the raw materials had such similar chemical compositions that they could be considered to be from the same raw material (or even from the same/analogous process). The distribution of the elements in the microstructure is illustrated by maps of the occurrences of the arsenic and lead in the copper matrix. It should be noted that the raw copper material did not contain intentional alloying additives and should, therefore, be considered to be virgin raw material.

The EBSD analyses showed that the primary copper grains were large (the maximum measured Cu grain diameter was 9598.2 µm), which indicated a slow crystallisation process that occurred in a low thermal conductivity form, (i.e., with slow heat dissipation from the solidifying ingot). Given their grain size as well as their irregular shapes and uneven edges, the ingots may have been cast into makeshift sand moulds.

The remaining pieces of the raw material hoard belong to the Cu-Sn or Cu-Sn-Pb tin-lead bronzes.

The axe with a socket (Prz.184a) was made using the casting technique in a two-part solid or semi-permanent mould with a core. The visible cavities were machined, and the macroscopic observations provided evidence of finishing and sharpening. This indicated that the axe originally had a utilitarian function which was lost as a result of the mechanical deterioration of the ear, perhaps due to an internal defect in the material. The material was tin-lead bronze with good casting and technological properties. The microstructure and chemical analysis confirmed the presence of solid solution, eutectoid, and intermetallic phases. Note the presence of copper sulphides in the microstructure; these were traces of the origin of the raw copper material from sulphide ores and the metallurgical process to which it was subjected, involving the roasting of sulphide ores.

Two fragments of a necklace (Prz.184b), which formed part of a necklace, were made using the casting technique. It was noted that the surface of the necklace was decorated, indicating that the necklace had been used and was damaged as a result of an internal cavity that was revealed at the fracture site. The necklace was cast from tin-lead bronze with a higher lead content than that of tin. The alloy composition also contained elements that were related to the origin of the raw copper material (Bi, As, Sb, Ag, Ni, and Zn) that, along with the lead, formed numerous intermetallic phases in the microstructure.

The fragment of a twisted-shaft necklace (Prz.184c; Figure 12 and Figure 13) was mechanically damaged. The necklace was decorated with regular parallel rows along its length, which invites discussion about its manufacture. Observations of the surface may suggest that the decoration was made on a wax model and the finished pattern was cast (so-called pseudo-ordering). However, the macroscopic analysis of the crack and the analysis of the microstructure clearly indicated that a process of plastic moulding and heat treatment (i.e., the twisting of the shank around an axis) was carried out. The characteristic crack was the result of the failure that occurred as a result of the stresses that developed in the structure during the process of the torsion of the rod and when bending it into the hoop. The torsion of the rod occurred when two pairs of forces acted in two different planes away from each other and perpendicularly to the rod axis; torsional moment was then created. Because there were no volume changes in the bar (only shape changes), it can be assumed that the stress state in the twisted bar was similar to a pure shear state. There were shear stresses in the cross-sections of the bar.

The necklace was originally cast as a bronze bar with only one alloy addition (in the form of tin) and a small proportion of natural elements (such as Pb, As, Ni, Fe, Zn, and copper sulphides [Cu_2_S]). The low lead content may have been natural or an indication that the bronze was from recycled scrap bronze that contained lead. The bar was then shaped to the appropriate cross-section and twisted in several successive operations. Traces of plastic and heat treatment were visible in the grain pattern of the structure. The cold forging of the tin bronze resulted in plastic deformation in the microstructure, which was characterised by deformation lines, followed by grain deformations and inclusions. Long-term cold treatment by forging led to a gradual strengthening of the material; eventually, the deformability of the artefacts decreased. Because the material became too brittle for further processing, it needed to be annealed in order to make it workable again.

The three necklace fragments (Prz.184d; Prz.184e; Prz.184f, Figure 14a) were cast in bar form and then worked. The pieces showed traces of incisions that were made with a chisel in order to divide the necklace (Figure 14d). The microstructure showed numerous cracks that resulted from the bending of the necklace coil. The microstructure also showed elongated precipitations and defects at the grain boundaries, which indicated plastic and heat-treatment processes (Figure 15). The microstructure showed the presence of numerous fine lead precipitations as well as copper and iron sulphides (Figure 17).

The chemical compositions of the necklace fragments were very similar. The fragments marked Prz.184d and Prz.184e had the same chemical profile as tin bronze, which confirmed that they belonged to a single ingot. The third fragment (Prz.184f) differed slightly in its proportions of tin and lead, as well as bismuth. The tin was a deliberate alloying addition, while the small amount of lead should be considered to be the result of the melting of the bronze raw material that contained lead. The microstructure of the Cu-Sn alloy showed residual copper and iron sulphides as well as lead precipitations.

### 4.2. Test Results of Model Alloys

We assumed that our observations and analytical tests would provide important information about the original artefacts while also allowing us to use the data in the experiment of casting model alloys that corresponded to the chemical profiles of the selected objects. These comprised two necklaces; namely, a necklace that was crafted using a casting technique (Prz.184b), and a twisted-shaft necklace (Prz.184c). The theoretical data that were obtained through the thermodynamic analysis that was carried out in the Thermo-Calc program were verified during the thermal analyses that were carried out during the crystallisations of the model alloys.

Alloy properties such as hardness, tensile strength, and elongation were also determined. A very important technological parameter of the alloy was determined, which was the funnelling of the alloy.

In the conducted experimental melting of the model alloys, the intended goals were achieved as to the obtained chemical compositions. Information was obtained on the influence of alloying elements such as tin, lead, bismuth, and arsenic on the properties of the alloys, with special attention being paid to the thermodynamic parameters of the crystallising alloys.

The TDA analysis that was performed and the Thermo-Calc modelling made it possible to determine the actual solidification range of the model alloys that were studied. The analysis of the crystallisation process in relation to the present and previous variants of the experiment that were, conducted without arsenic Should be [59,60], showed that even a small amount of a characteristic element (arsenic in this case) changed the nature of the crystallisation- lowering the temperatures at the beginnings and ends of the alloy solidifications.

The CuSn-type alloy had a greater solidification range than the CuPbSn-type alloy, which was reflected in the result of the technological parameter—alloy flowability. The CuSn-type model alloy (Prz.184c) had higher tensile strength and alloy hardness as well as slightly lower elongation when compared to the CuPbSn-type alloy (Prz.184b). Mainly, the achieved strength parameters of the alloys were affected by the addition of tin; higher tin contents induced thes hardening of the alloys.

## 5. Conclusions

Due to the limited scope of non-destructive analysis in archaeometallurgy, it is necessary to constantly search for new, alternative, and increasingly accurate research methods. In the proposed methodology, it is important to experimentally reproduce ancient alloys and technologies without damaging original materials in order to lead to new data. Macroscopic and microscopic observations can confirm the diversity of old manufacturing techniques, including both the casting and plastic-working techniques.

Analyses of the technology behind the various elements of the find allowed the hoard to be assessed as a deliberately differentiated raw material deposit (of measurable economic value) which was made during an act of symbolic offering.

The study of the Przybysław hoard has shown that we are dealing with an interesting and rare case of a raw material hoard that includes primary raw copper material (preformed), raw iron material, and bronze scrap. It should be noted that the raw copper material that was contained in the treasure is was a unique find. It was not alloyed with tin, so it can be considered to be primary raw material; also, the accompanying components were natural elements from the ore that remained in the microstructure as a result of the smelting from the ore. This fact may indicate the existence of a local production centre that obtained raw material from distant ore mines.

By examining the nature of the compositions and structures of the artefacts that are included in the hoard, making detailed assessments about how each individual artefact was treated prior to its inclusion in the hoard, and determining what caused its destruction, it became clear that their inclusion in the hoard was not coincidental, as their association with the material defects and metalworking processes was recognised.

It was significant (and certainly intentional) that each object was damaged and, once the fragments were assembled, none was a whole. Also intentional was the diversity of the hoard in terms of the chemistry profile of its components and the manufacturing techniques that were used to produce them.

In the case of the Przybysław hoard, all of the bronze objects that were discovered in it (an axe and fragments of necklaces) showed the presence of primary or secondary damage, which contributed to their removal from circulation.

The artefacts that were already damaged during the technological process itself (Prz.184a—axe; Prz.184c—twisted-shaft necklace) or those in which internal defects may have contributed to their damage (Prz.184b—cast necklace) were destined for the hoard. The elements of the Prz.184 d-f necklace are the only ones that appear to have been deliberately destroyed, and traces of the tool (a chisel) with which the destruction was carried out can be seen on a fragment of the Prz.184d necklace.

Studies into the chemical compositions of the bronze scrap have identified the intentionally introduced alloying elements as well as those elements that resulted from the composition of the ore.

The results of the research not only provided important information about the objects themselves but also allowed these data to be used in the thermodynamic-modelling process as well as in the experiment that was carried out on the model alloys. In this way, new data on the properties of the selected alloys could be obtained using a non-destructive method.

On the basis of extensive observation and research, it has been shown that the hoard was technical in nature and composed of raw materials and bronze scrap that linked the foundryman’s and blacksmith’s workshops, and that the objects that were deposited in the Przybysław hoard were deliberately selected, fragmented, and excluded from circulation due to metallurgical behavioral context and left in safekeeping as a ritualistic act of offering.

## Figures and Tables

**Table 1:** XRF chemical composition of copper raw materials and bronze products from the raw material hoard (wt.%).

Element	Fe	Co	Ni	Cu	Zn	As	Ag	Sn	Sb	Pb	Bi
Prz.184i.R1	0.07	0.05	0.10	97.93	0.18	0.21	0.12	0.03	<0.05	1.01	0.30
Prz.184i.R2	0.10	0.07	0.10	97.40	0.14	0.40	0.15	0.02	<0.05	1.25	0.37
Prz.184i.R3	0.14	0.07	0.10	97.90	0.16	0.27	0.11	0.10	<0.05	0.90	0.25
Prz.184a	<0.025	0.08	0.48	90.50	0.14	0.37	0.22	5.32	1.00	1.87	0.03
Prz.184b	0.03	0.08	0.13	89.73	0.13	0.32	0.09	2.37	0.09	6.44	0.59
Prz.184c	0.11	0.11	0.12	90.10	0.15	0.27	0.02	8.49	<0.051	0.56	0.07
Prz.184d	0.12	0.12	0.13	89.75	0.14	0.24	0.04	8.39	0.08	0.91	0.08
Prz.184e	0.10	0.11	0.13	90.04	0.14	0.21	0.04	8.21	0.03	0.92	0.07
Prz184f	0.16	0.14	0.14	88.88	0.16	0.36	0.05	8.71	<0.05	1.42	0.16

**Table 2:** EDS chemical composition of the Prz.184i.R2 ingot for Figure 20 (wt.%).

Conc.	Cu	As	Sn	Sb	Pb	Bi
R2_pt1	13.79	12.73	-	-	73.49	-
R2_pt2	16.55	14.06	-	2.12	67.27	-
R2_pt3	14.64	11.88	-	1.36	65.82	6.30
R2_pt4	12.90	11.54	2.40	1.47	71.70	-
R2_pt5	18.51	11.00	-	-	70.49	-
R2_pt6	100.00		-	-	-	-

**Table 3:** EDS chemical composition of the Prz.184i.R2 ingot for Figure 21 (wt.%).

Conc.	Cu	As	Ag	Pb	Bi
R2_pt1	23.32	-	17.65	-	59.03
R2_pt2	11.17	12.56	-	-	76.27
R2_pt3	16.97	14.28	-	68.75	-
R2_pt4	100.0	-	-	-	-

**Table 4:** Phases in the Prz.184i.R3 ingot (by EBSD) in the micro-area from Figure 26.

Phase Number	Phase Name	Phase Fraction (%)	Phase Count	Crystal System
1	Copper	97.00	190,705	Cubic
2	Cu_2_O	0.55	1087	Cubic
3	PbS	0.08	161	Cubic
4	AsS	0.05	103	Monoclinic
5	Zero Solutions	2.32	4407	

**Table 5:** EDS chemical composition of the Przy.184a axe for Figure 30 (wt.%).

Conc.	Cu	Sn	Pb
Prz184a_pt1	61.41	17.78	20.81
Prz184a_pt2	11.80	34.64	53.56
Prz184a_pt3	94.63	5.37	-

**Table 6:** EDS chemical composition of the Przy.184a axe for Figure 31 (wt.%).

Conc.	S	Fe	Cu	Sn	Pb
Prz184a_pt1	19.65	1.85	78.50	-	-
Prz184a_pt2	-	-	51.49	4.33	44.18
Prz184a_pt3	-	-	95.22	4.78	-

**Table 7:** EDS chemical composition of the Przy184b necklace for Figure 32 (wt.%).

Conc.	O	Cu	Sn	Pb
Prz184ba_pt1	-	92.77	2.72	4.52
Prz184ba_pt2	-	94.52	0.00	5.48
Prz184ba_pt3	-	80.01	0.00	19.99
Prz184ba_pt4	-	36.65	3.94	59.41
Prz184ba_pt5	15.39	84.61	0.00	0.00
Prz184ba_pt6	-	100.00	0.00	0.00

**Table 8:** EDS chemical composition of the Przy184c necklace for Figure 33 (wt.%).

Conc	Cu	Sn	Pb
Prz184c_pt1	67.65	7.18	25.17
Prz184c_pt2	82.19	7.11	10.71
Prz184c_pt3	92.66	7.34	-

**Table 9:** EDS chemical composition of the Przy184c necklace for Figure 34 (wt.%).

Conc	S	Cu	Sn	Pb
Prz184c_pt1	-	5.35	0.00	94.65
Prz184c_pt2	6.22	92.27	1.51	-
Prz184c_pt3	-	93.86	6.14	-

**Table 10:** EDS chemical composition of the Przy184d necklace for Figure 35 (wt.%).

Conc.	S	Cu	Sn	Pb
Prz 184 d_pt1	-	38.90	26.36	34.74
Prz 184 d_pt2	4.25	83.41	12.33	-
Prz 184 d_pt3	-	92.26	7.74	-

**Table 11:** EDS chemical composition of the Przy184e necklace for Figure 36 (wt.%).

Conc.	S	Fe	Cu	Sn	Pb
Prz184e_pt1	-	-	12.71	-	87.29
Prz184e_pt2	9.08	1.58	31.90	-	57.44
Prz184e_pt3	16.51	1.92	79.19	2.38	-
Prz184e_pt4	-	-	90.43	9.57	-
Prz184e_pt5	-	-	89.96	10.04	-

**Table 12:** Chemical composition of the Prz.184f necklace for Figure 37 (wt.%).

Concentration	S	Fe	Cu	Sn	Pb
Prz184f_pt1	-	-	23.54	-	76.46
Prz184f_pt2	22.78	5.84	71.38	-	-
Prz184f_pt3	22.86	4.59	72.54	-	-
Prz184f_pt4	-	-	82.33	6.59	11.08
Prz184f_pt5	-	-	90.35	9.65	-
Prz184f_pt6	-	-	87.00	10.78	1.85

**Table 13:** Chemical compositions of the model alloys that were prepared in the experiment for the Prz.184b and Prz.184c necklaces, which corresponded to the compositions in Table 1 (wt%).

No.	Fe	Co	Ni	Cu	Zn	As	Ag	Sn	Sb	Pb	Bi
	(wt.%)										
**Alloy 1.** CuPbSn (Prz.184b)	**0.03**	**0.08**	**0.13**	**89.73**	**0.13**	**0.32**	**0.09**	**2.37**	**0.09**	**6.44**	**0.59**
(1) CuPb6.5	0.001	0.002	0.003	93.3	0.02	0.006	0.001	0.07	0.03	6.52	0.01
(2) CuPb6Sn2.2	0.001	0.001	0.002	91.8	0.014	0.006	0.001	2.22	0.02	5.91	0.004
(3) CuPb6Sn2.3Bi0.3	0.001	0.001	0.002	91.3	0.012	0.006	0.001	2.32	0.02	6.10	0.28
(4) CuPb6.5Sn2.4Bi0.8As0.3	0.01	0.001	0.133	89.28	0.106	0.34	0.129	2.54	0.10	6.54	0.81
**Alloy 2.** CuSn (Prz.184c)	**0.11**	**0.11**	**0.12**	**90.10**	**0.15**	**0.27**	**0.02**	**8.49**	**<0.051**	**0.56**	**0.07**
(1) CuSn8Pb0.7As0.3Zn	0.07	0.001	0.131	90.51	0.24	0.28	0.001	8.00	0.02	0.69	0.07

**Table 14:** Characteristic temperatures of crystallisation were determined on basis of TDA for the CuPbSn alloy casting experiment.

Sample	T_1_		T_2_		T_3_		T_4_		T_5_	
							[°C]			
	TDA	TC	TDA	TC	TDA	TC	TDA	TC	TDA	TC
CuPb6.5	1048.9	1062.0	951.0	946.0	323.6	326.0	-	-	-	-
CuPb6.5Sn2.4	1041.2	1045.0	922.1	918.0	314.2	326.0	299.7	-	-	132.0
CuPb6.5Sn2.4Bi0.3	1034.8	1044.0	914.4	916.0	289.0	313.0	-	301.0	-	133.0
CuPb6.7Sn2.6Bi0.6As0.3	-	1042.0	-	908.0	-	313.0	-	301.0	-	138.0
CuPb6.5Sn2.3Bi0.8As0.3	1022.8	1038.0	904.2	898.0	286.1	293/286	266.0	270.0	-	-

**Table 15:** Examples of TC modelling and TDA analysis for the Prz_184b and CuPbSn alloys.

Sample	T_1_	T_2_	T_3_	T_4_	T_5_	T_6_	T_7_	Mass Percent at 20 °C
	[°C]							[%]
TDA	1022.83	904.23	-	-	286.10	266.03	-	-
LIQUID	1038.13	898.66	-	-	-	263.96	-	-
FCC_L12	1038.13	898.66	-	-	-	-	20	86.76
FCC_L12#2	-	-	-	-	293.58	-	20	7.24
FCC_L12#3	-	-	-	-	-	270.18	20	0.10
BCC_B2	-	-	-	539.94	-	-	20	0.11
BCC_B2#2	-	-	584.31	539.94	-	-	-	-
CU3SN	-	-	-	-	286.16	-	20	5.79
CU10SN3	-	-	-	408.55	286.16	-	-	-

**Table 16:** Examples of TC modelling and TDA analysis for the Prz.184c (CuSn8Pb0.7As0.3Zn) alloys.

Sample	T_1_	T_2_	T_3_	T_4_	T_5_	T_6_	Mass Percent at 20 °C
	[°C]						[%]
TDA	1010.84	754.22	573.91	509.10	278.77		-
LIQUID	1017.66	788.00	-	-	263.83		-
FCC_L12	1017.66	788.00	-	-	-	20	78.07%
BCC_B2#2	-	-	630.42	-	-	20	0.13%
CU3SN	-	-	-	470.36	-	20	21.17%
FCC_L12#2	-	-	-	-	292.43	20	0.63%

**Table 17:** Crystallisation range that was determined by TDA method on the basis of the conducted experiments of tested model alloys.

Sample	T1 [°C]	T2 [°C]	Actual Range of Solidification
CuPb6.5Sn2.3Bi0.8As0.3 (184b)	1022.83	898.7	118.6
CuSn8Pb0.7As0.3Zn (184c)	1010.84	754.22	256.6

**Table 18:** Results of the tensile strength (UTS), elongation (A), hardness (HBS), and alloy flowability tests for the assessed CuPb6.5Sn2.3Bi0.8As0.3 (Prz.184b) and CuSn8Pb0.7As0.3Zn (Prz.184c) alloys.

Sample	Metal Mould			Sand Mould	
	UTS [MPa]	A [%]	HBS	HBS	Alloy Flowability [m]
Alloy 1. CuPb6.5Sn2.3Bi0.8As0.3 (Prz.184b)	240.4	32.1	83.2	68.1	0.55
Alloy 2. CuSn8Pb0.7As0.3Zn (Prz.184c)	280.5	22.0	108.5	93.1	1.15

## Data Availability

The data that support the findings of this study are available from the corresponding authors (A.G.-K., P.S., M.S., M.W.-L., J.K., M.P., M.P.-N.) upon reasonable request.
